# The Sleeping Beauty: How Reproductive Diapause Affects Hormone Signaling, Metabolism, Immune Response and Somatic Maintenance in *Drosophila melanogaster*

**DOI:** 10.1371/journal.pone.0113051

**Published:** 2014-11-13

**Authors:** Olga I. Kubrak, Lucie Kučerová, Ulrich Theopold, Dick R. Nässel

**Affiliations:** 1 Department of Zoology, Stockholm University, S-106 91 Stockholm, Sweden; 2 Department of Molecular Biosciences, Wenner-Gren Institute, Stockholm University, Stockholm, Sweden; Ecole Normale Supérieur de Lyon, France

## Abstract

Some organisms can adapt to seasonal and other environmental challenges by entering a state of dormancy, diapause. Thus, insects exposed to decreased temperature and short photoperiod enter a state of arrested development, lowered metabolism, and increased stress resistance. *Drosophila melanogaster* females can enter a shallow reproductive diapause in the adult stage, which drastically reduces organismal senescence, but little is known about the physiology and endocrinology associated with this dormancy, and the genes involved in its regulation. We induced diapause in *D. melanogaster* and monitored effects over 12 weeks on dynamics of ovary development, carbohydrate and lipid metabolism, as well as expression of genes involved in endocrine signaling, metabolism and innate immunity. During diapause food intake diminishes drastically, but circulating and stored carbohydrates and lipids are elevated. Gene transcripts of glucagon- and insulin-like peptides increase, and expression of several target genes of these peptides also change. Four key genes in innate immunity can be induced by infection in diapausing flies, and two of these, *drosomycin and cecropin A1*, are upregulated by diapause independently of infection. Diapausing flies display very low mortality, extended lifespan and decreased aging of the intestinal epithelium. Many phenotypes induced by diapause are reversed after one week of recovery from diapause conditions. Furthermore, mutant flies lacking specific insulin-like peptides (*dilp5* and *dilp2-3*) display increased diapause incidence. Our study provides a first comprehensive characterization of reproductive diapause in *D. melanogaster*, and evidence that glucagon- and insulin-like signaling are among the key regulators of the altered physiology during this dormancy.

## Introduction

A capacity for adaptive changes in response to environmental challenges is critical for animal survival. Most environmental stresses vary over time and space and resistance may be acquired by adaptive life history traits. Thus, when exposed to harsh conditions animals, such as for instance insects and nematode worms, enter a reversible state of developmental arrest and metabolic restructuring, coupled with increased stress resistance and extended lifespan [1], [2], [3], [4], [5], [6], [7]. In many insects this temporary suppression of development and reallocation of energy resources is pre-programmed and obligate known as diapause or dormancy [4], [5]. In other insects, such as *Drosophila melanogaster*, diapause is facultative, appears not to be triggered by photoperiodic signals, and the dormancy is more shallow and reversible, [8], [9], [10], [11], [12]. Nevertheless, the dormancy in *D. melanogaster* has been referred to as diapause by most authors.

In *D. melanogaster* reproductive diapause can be induced in adult females by a combination of low temperature and short day length [13], [14], [15]. Diapausing flies do not develop vitellogenic eggs, and display slowed organismal senescence and extended longevity [13], [14], [16], [17]. Adult female *D. melanogaster* that had been kept in reproductive diapause for up to nine weeks and then transferred to non-diapausing conditions were found to age at the same rate as newly eclosed non-diapause control flies [14]. Thus, time in reproductive diapause seem to pass with the fly's physiology and metabolism running at very basal levels and with a drastically reduced senescence.

One proposed key regulator of diapause induction and maintenance is insulin/IGF signaling (IIS) [5], [14], [18], [19], [20]. IIS also regulates development, metabolism, fecundity, stress resistance and lifespan [21], [22], [23], [24], [25], all of which are likely to be affected by diapause. We are interested in unraveling the organismal changes and regulatory mechanisms underlying the slowed metabolism, increased stress resistance and halted senescence during diapause.

Although a number of insect species have been studied in some detail with respect to endocrine control of diapause and diapause-induced alterations of gene expression, physiology and metabolism [4], [5], [11], [26], [27], [28], these aspects have received little attention in *D. melanogaster*. This is surprising since *D. melanogaster* is a widely explored model organism for analysis of adaptations in metabolism and energy homeostasis [29], [30], [31], [32], as well as in studies of fecundity, stress responses and regulation of lifespan [23], [33], [34]. Thus, it would seem that *D. melanogaster* is an excellent organism for studying the genetics, endocrinology and metabolism of reproductive diapause. We therefore monitored effects of reproductive diapause on feeding, carbohydrate and lipid metabolism, as well as gene expression associated with insulin-like (DILP) and glucagon-like (adipokinetic hormone; AKH) signaling and innate immunity. Furthermore we monitored effects of diapause on morphology of the intestine since this is known to change with feeding rate and aging. We investigated two commonly studied *D. melanogaster* strains, *Canton S* and *w^1118^*, and find that the former appears more prone to enter diapause and is affected more drastically by diapausing conditions in our assays. Thus, we use *Canton S* for the core of the investigation and show *w^1118^* data for comparison since it is a fly strain often employed as a control in experiments.

Since insulin signaling plays an important role in diapause [5], [12], [18], [19], [22], [35], [36] we also analyzed two strains with deficiencies in insulin-like peptides. In *D. melanogaster* there are eight DILPs (DILP1-8) and one receptor tyrosine kinase (dInR) that act in an evolutionarily conserved signaling pathway [21], [25], [33], [37], [38], [39]. It is known for *D. melanogaster* that a natural polymorphism in a component of the insulin signaling pathway, phosphatidylinositol 3-kinase (PI3K), affects tendency to enter reproductive diapause [20]. Thus, we analyzed the effect on diapause of impaired insulin signaling by using mutant strains with loss of function of DILP2 and 3 or DILP5 [21]. These three DILPs are known to be expressed by the brain insulin-producing cells, IPCs and appear sufficient for regulation of stress resistance, fecundity, metabolic homeostasis and lifespan [23], [24], [25], [30], [40].

We obtained compelling evidence for a shallow diapause phenotype in *D. melanogaster*, with drastically decreased food intake, altered levels of circulating and stored carbohydrates, upregulated *dilp* and *Akh* transcripts, as well as two Toll-related innate immunity genes, and slowed aging of the midgut epithelium. Furthermore, we detected decreased incidence of diapause induction in flies with deficiencies in insulin signaling due to mutations in *dilp5* or *dilp2* and *dilp3* genes.

## Results

### Induction of reproductive diapause by short day length and low temperature: phenotyping by ovarian development

Although some authors consider that only low temperature, and not day length (photoperiod), is critical for induction of adult reproductive diapause (see [5], [14]) in *D. melanogaster*, we decided to follow the protocol of Saunders for the *Canton S* strain [13], [41] and Tatar for the *Windsor* strain [14] and expose virgin female flies to a combination of 11°C and short photoperiod (10L:14D). Thus, in our experiments we used newly eclosed 3–6 h old virgin wild type flies (*Canton S* strain) or other strains (as specified later) for experiments. These 3–6 h flies (designated C0) were placed in incubators under diapause conditions and sampled for a number of assays, including analysis of ovaries, every week for the first three weeks and thereafter after 6, 9 and 12 weeks (these are labeled D1–D12). For comparison in each assay we used virgin flies kept for one week under non-diapause conditions (12L:12D at 25°C) and designated C1 in figures. These one week-old non-diapausing flies should also correspond phenotypically to flies used as controls in studies of insulin signaling and metabolism in *Drosophila*. We furthermore assayed virgin flies that had recovered at 25°C and 12L:12D for 1 week after 3 weeks of diapause (R1′) and for 1 week (R1) or 2 weeks (R2) after 6 weeks of diapause.

**Figure 1:**
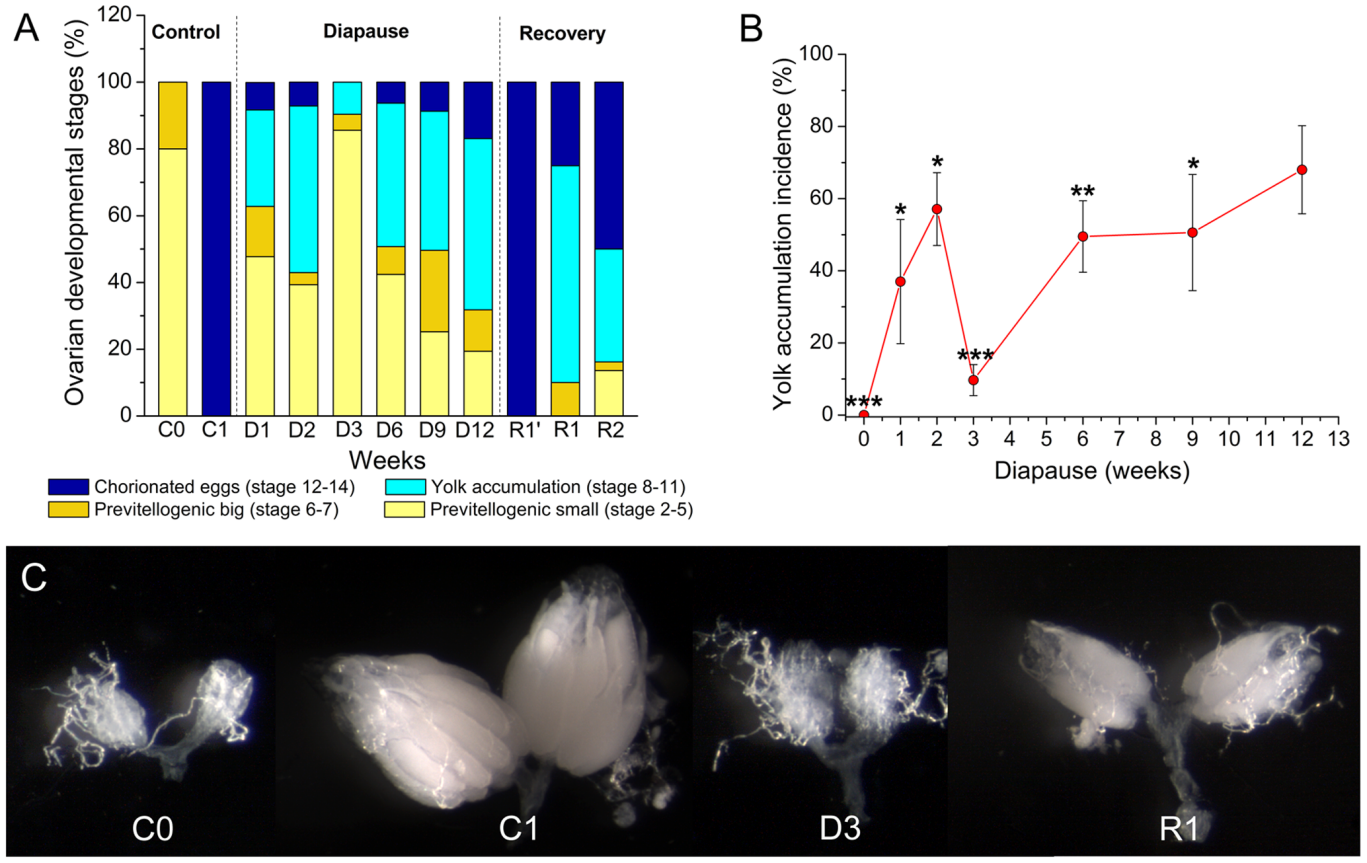
Ovarian development and yolk accumulation are arrested during diapause. **A** Ovarian developmental stages (%) in virgin female flies *Drosophila melanogaster Canton S* (A), kept for 1–12 weeks at 11°C and short photoperiod 10L:14D (diapause, D1–D12) or after recovery for 1 week after 3 weeks of diapause (R1′), 1 (R1) or 2 weeks (R2) after 6 weeks of diapause. Recovery was carried out at 25°C and normal photoperiod 12L:12D, light/dark. As comparison we used 3–6 h old (newly eclosed) virgin flies (C0) or virgin flies kept under normal conditions for 1 week (C1). The developmental stage of ovaries was assessed as outlined in Material and methods. Note that one week control flies (C1) have fully developed ovaries. Data are presented as means ± S.E.M, *n* = 4 independent replicates with 8–12 flies in every replicate. **B** Yolk accumulation incidence (%) in ovaries of virgin female flies *(Canton S)* kept at the same conditions as in 1A. 1-week old flies, kept under normal conditions (C1) were used as reference. Yolk accumulation incidence (%) was defined as the presence yolk deposit even in a single oocyte (stage 8) up to formation of one/several chorionated eggs (stages 12–14) in mostly previtellogenic ovaries of diapausing flies. Data are presented as means ± S.E.M, *n* = 4 independent replicates with 8–12 flies in every replicate. We indicate data that are significantly different from the C1 flies kept for one week at normal conditions (C1) that display 100% yolk incidence (Student *t*-test with *p<0.05, **p<0.01, ***p<0.001). See Fig. S1 in File S1 for ovary development in *dilp* mutants and Fig. S6A, B in File S1 for *w^1118^* flies. **C** Representative images of ovaries at selected stages of *Canton S*.

The percentage of vitellogenic ovaries is commonly used for phenotyping reproductive diapause in *Drosophila* [8], [13], [15], [42], [43]. Thus, to establish the time course and incidence of reproductive diapause we monitored the status of ovarian development by using a combination of criteria established earlier [13], [44], [45], each week in flies kept in diapausing conditions, as well as in control flies kept at 25°C and 12L:12D for one week. We provide a detailed description of the staging we use in Material and methods (see also Fig. 1A), since earlier reports were not clearly defining what constitutes diapausing ovaries.

*D. melanogaster* is known to lack inter-ovariole synchrony (King, 1970), and this may be enhanced under diapause. Therefore we monitored all stages in ovarian development, from fully previtellogenic ovaries to a presence of several chorionated eggs, in virgin *Canton S* flies, kept under diapause conditions (Fig. 1A). There are high percentages (37–62%) of yolk accumulation incidence the first 1–2 weeks (D1–D2) (Fig. 1B). However, after 3 weeks of diapause (D3) the percentage of eggs that have accumulated any traces of ovarian yolk is at a minimum (less than 10%) (Fig. 1A–B). Thus, the ovaries in flies kept in diapause for 3 weeks are the least vitellogenic and resemble those in newly emerged control (C0) flies (Fig. 1C), although the latter have only previtellogenic ovaries (Fig. 1A–B). Over weeks 6–12 of diapause (D6–D12) vitellogenesis is slightly higher and there is an increased number of ovaries at stages 8–12 (Fig. 1A), as well as elevated yolk accumulation incidence (Fig. 1B). More specifically the incidence of ovaries displaying yolk accumulation is about 50% during 6 and 9 weeks of diapause, but after 12 weeks an increase up to 70% is noted (Fig. 1B). Transfer of flies, from 3 weeks of diapause conditions to normal conditions for one week (recovery, R1′) leads to formation of ovaries with several chorionated eggs (Fig. 1A). However these flies do not display the full ovarian development that can be observed in the 1-week old flies (C1) kept under normal conditions (Fig. 1A, C). Recovery from 6 weeks of diapause was less complete than after 3 weeks of diapause. Even after 2 weeks of recovery (R2) vitellogenesis is not complete in all flies and some previtellogenic ovaries can still be found (Fig. 1A). Thus, having established that the temperature and light regime used indeed induces reproductive diapause in wild type flies, we applied it in experiments investigating further aspects of diapause in *D. melanogaster*.

### Feeding and body mass changes during diapause

Since diapause in *D. melanogaster* occurs in the adult stage it is likely that the flies feed. However, to our knowledge there are no published reports on food ingestion in diapausing fruitflies. Hence, we quantified food intake during diapause as follows. We collected flies at different times of diapause similar to those investigated for ovary development and allowed them to feed on a food-dye mixture for 6 h. After feeding the flies were homogenized and dye-ingestion determined by spectrophotometric quantification (see [46], [47]). Food intake in flies kept for 1 week under non-diapausing conditions (C1) was used as a comparison.

**Figure 2:**
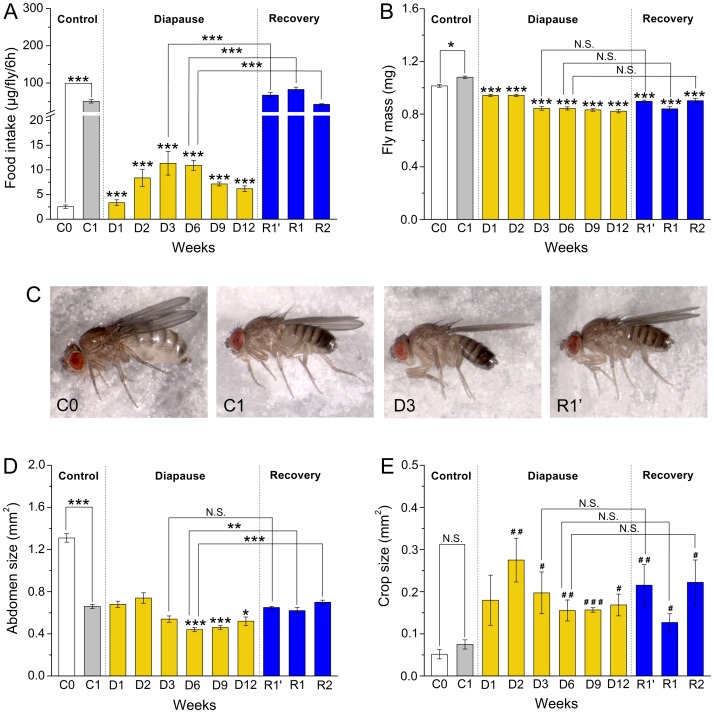
Food intake and body mass are reduced during diapause in *D. melanogaster (Canton S)*. **A** Food intake during 6 h was measured in flies of *Canton S* strain at different time points by feeding flies dyed food (measured as µg/fly/6 h). We used 1 week old (C1) virgin flies in non-diapause conditions as comparisons. Flies were kept for 1–12 weeks in diapause conditions at 11°C and 10L:14D (D1–D12). We also tested flies (blue bars) placed for 1 and 1–2 weeks at 25°C and 12L:12D after either 3 weeks (R1′) or 6 weeks (R1, R2) of diapause. During diapause food intake decreases drastically already after one week, but increases slightly to a peak after three weeks. Food consumption reverts back to non-diapausing levels after recovery from diapause. Data are presented as means ± S.E.M, *n* = 5–6 independent replicates with 6–10 flies in each replicate. We indicate significance values for experimental flies compared to the 1 week non-diapausing control (C1, grey bar) or as indicated by connectors (***p<0.001, as assessed by ANOVA followed with Tukey test). **B** Body mass of whole flies was measured in flies corresponding to the sampling points in 2A. A significant decrease of body mass is seen already after one week of diapause (D1) compared to C1 controls, but no significant gain is seen after recovery from diapause (R1′, R1–R2). Data are presented as means ± S.E.M, *n* = 8–10 independent replicates with 10–15 flies in each replicate. Statistics as in Fig. 2A (*p<0.05, ***p<0.001). See Fig. S4 and S7C, D in File S1 for comparisons of food intake and body mass in *dilp* mutants and *w^1118^*. **C** Representative images of flies at different stages: 3 h normal conditions (C0), 1 week normal conditions (C1), 3 weeks diapause (D3), and 1 week of recovery after one week diapause (R1′). **D** Abdomen size (mm^2^) of female *Canton S* flies, kept under conditions as in A2. The largest abdomens are found in the newly eclosed flies (C0), which are full of pupal fat body. In flies, kept under diapause conditions for 6–12 weeks (D6–D12) the abdomen size is decreased. After recovery from diapause the abdomen size increases and resembles that of 1-week old control flies (C1) kept at non-diapausing conditions. Statistics as in Fig. 2A (*p<0.05, **p<0.001, ***p<0.001). **E** The crop size increases during diapause with a maximum after 2 weeks, and remains larger than the C1 control during 12 weeks. See Fig. S1 in File S1 for images of crops. We indicate significance values for experimental flies compared to the 1 week non-diapausing control (C1, grey bar) or as indicated by connectors (^#^ *p*<0.05, ^##^ *p*<0.01 or ^####^ *p*<0.001 as assessed by Kruskal–Wallis test followed by pairwise comparisons using Wilcoxon rank sum test).

Food consumption was very small in 3–6 h old flies (C0) compared to one-week-old flies kept under non-diapause conditions (C1) (Fig. 2A). This is in line with a previous study [48]. Compared to non-diapausing flies (C1) the 2–12 week diapausing flies (D1–D12) ingest drastically less food, although they feed more than newly eclosed flies (C0) (Fig. 2A). A small peak in food intake (13 µg/fly/6 h) is seen at 3 weeks of diapause (Fig. 2A). This can be compared to one-week-old non-diapausing flies, which consume about 50 µg/fly food during 6 h (Fig. 2A). Flies that had recovered from 3 or 6 weeks diapause (R1′, R1–R2) resumed feeding at a level similar to that of non-diapausing C1 flies, and about 4–5 times more than diapausing ones (Fig. 2A). Overall, diapausing flies thus feed at a very low level, but are able to resume their normal food intake after one week of recovery in non-diapausing conditions.

We determined the body mass of flies kept under the same regimes as above. Diapausing flies display significantly decreased weight (13–24% reduction) compared to 1-week-old flies (C1) kept under non-diapause conditions (Fig. 2B). Especially flies kept for 3–12 weeks in diapause conditions have reduced weights (more than 20% reduction). Recovery from diapause did not lead to a significant gain in body mass (Fig. 2B). In summary, diapausing flies display reduced body mass probably due to decreased food intake, and increased feeding during recovery from diapause does not compensate for this weight loss.

Next we monitored the size of the abdomen of the experimental flies and found that there was a significant decrease (by 18–34%) in 3–12 week diapausing flies and an increase after recovery (Fig. 2C, D). The 3–6 h old flies (C0) had significantly larger abdomens than all other flies. Finally we measured the size of the crop, a diverticulum from the foregut, used for temporal storage of nutrients [49]. We found that the crop was significantly dilated over 2–12 weeks of diapause, compared to 1-week non-diapausing flies (C1) and its size remained large after recovery (Fig. 2E, Fig. S1 in File S1). The maximal crop size (3.7-fold larger than in C1 flies) was seen at about 2 weeks of diapause. Furthermore the crop contents are translucent in diapausing flies, in contrast the opaque ones seen in non-diapausing and recovered flies (Fig. S1 in File S1). It can be noted that the peptide hormones DILP2 and AKH are thought to be responsible for crop filling/empting [49], [50], [51], [52] and thus alterations in signaling with peptides during diapause could affect crop size.

### Carbohydrate and lipid homeostasis is altered by diapause

It is known that metabolism and energy storage change drastically during, or in preparation for diapause [5], [53]. Thus we measured levels of circulating (hemolymph) and stored (body) carbohydrates as well as triacylglycerides (TAG) sampled as in the previous sections. For comparison we used virgin flies kept for one week under non-diapause conditions (C1). We also monitored total protein levels in hemolymph and whole body. It has to be kept in mind that the diapausing flies still feed at low levels and therefore we monitor a steady state carbohydrate and lipid metabolism that is lowered and dynamic, and not a simple consumption of stored nutrients as in developmental diapause.

**Figure 3:**
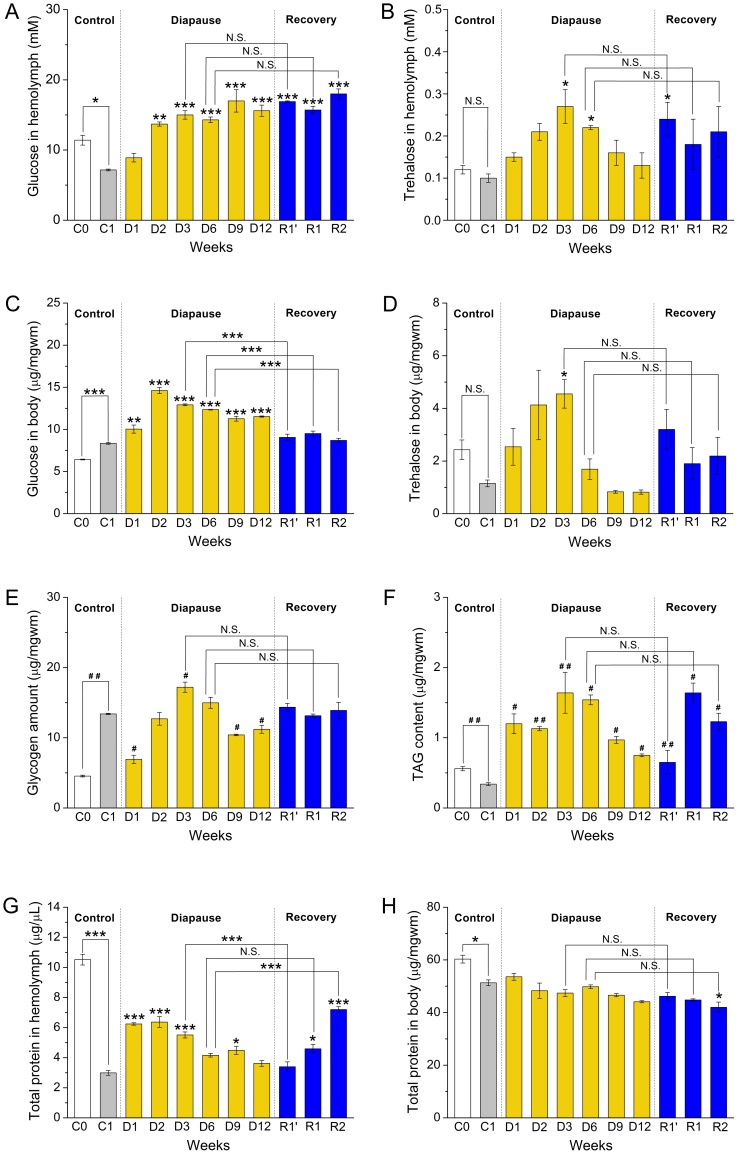
Diapause conditions affect circulating and stored carbohydrates and proteins as well as stored lipids in *Canton S*. Flies were kept under the same conditions as in Fig. 2A. In the panels data are presented as means ± S.E.M, *n* = 5–6 independent replicates with 10–15 flies in each replicate. Data significantly different from the flies kept for one week at normal conditions (C1) are indicated * *p*<0.05, ** *p*<0.01, ***p<0.001, N.S. not significantly different (ANOVA followed with Tukey test) or with ^#^ *p*<0.05, ^##^ *p*<0.01 or ^####^ *p*<0.01 (Kruskal–Wallis test followed by pairwise comparisons using Wilcoxon rank sum test). **A** Glucose concentrations in hemolymph (mM) are significantly increased throughout diapause as compared to the flies kept at normal conditions for one week (C1). After recovery from diapause (R1′–R2) glucose levels remain high. **B** Trehalose levels in hemolymph increase to a peak at three weeks of diapause (D3) and then return to the control level (C1). Recovery conditions have no effect on trehalose. **C** Whole body glucose also increases significantly during diapause, but falls after recovery compared to controls (C1). **D** Whole body trehalose stores increase significantly to a peak at three weeks diapause (D3) and drop thereafter to very low levels, similar to one week controls (C1). **E** Compared to the 1 week controls (C1) glycogen stores first drop (D1) and then increase with a peak at three weeks of diapause (D3). During recovery (R1′, R1 and R2) flies restore glycogen to the control (C1) value. **F** Triacylglycerid (TAG) contents increase significantly and also peak after three weeks of diapause (D3) and remain elevated compared to C1. **G** The total protein in the hemolymph is elevated during 1–3 weeks of diapause (D1–D3) compared to 1 week control flies (C1). After recovery from three weeks of diapause protein levels remain reduced, but after 2 weeks of recovery from 6 weeks of diapause proteins are elevated in the hemolymph. **H** The total body protein is similar to the one week non-diapausing controls (C1) throughout diapause. See also Fig. S5, S6 and S7E–L in File S1 for comparisons of metabolite levels in *dilp* mutants and *w^1118^*.

Glucose levels in the circulation are tightly regulated by DILPs and the glucagon-like peptide AKH [23], [24], [51], [54], [55] and possibly glucose homeostasis changes during diapause. We found that the hemolymph glucose concentration in one week control flies (C1) was reduced by 37% compared to the recently hatched C0 flies (Figure 3A), but similar to that of one week old diapausing flies (D1). However, starting from 2 and up to 12 weeks of diapause (D1–D12) hemolymph glucose levels increased 1.9–2.4 fold compared to C1 flies (Figure 3A). Over 3 to 12 weeks glucose levels were fairly constant with a trend towards maximum levels after 9 weeks. Transfer of flies from 3 or 6 weeks of diapause back to normal conditions (recovery; R1′, R1–R2) does not result in altered levels of circulating glucose compared to the corresponding diapausing flies, and glucose concentrations after recovery are above that of C1 controls (Figure 3A). Thus, the resumed feeding and possible hormonal changes after recovery does not affect circulating glucose within this time frame.

Next we monitored trehalose levels in the hemolymph. Trehalose is a disaccharide utilized both as an energy source and as a cryoprotectant [5], [35], and may play a role in stabilizing proteins and lipid bilayers during water deficit [56]. We found that circulating trehalose levels slowly increased in diapausing flies compared to non-diapausing controls (C1) and displayed a peak level at 3 weeks of diapause, where the hemolymph levels are 2–7 fold higher than that in C1 flies (Fig. 3B). After 9 and 12 weeks diapause trehalose levels were back to control levels. After recovery from diapause the trehalose levels were not different from corresponding diapausing flies.

Developmental diapause is usually associated with an accumulation of stored nutrients during a preparatory phase and then a gradual depletion of accumulated reserves during actual diapause [4], [5], [53], [57]. In *D. melanogaster* adult diapause is not programmed and thus there seems to be no *bona fide* preparatory phase. Furthermore, the flies continue to feed during diapause, which means that we may expect a different course of nutrient storage, compared to insects with programmed diapause. We monitored stored (whole body) glucose, trehalose, glycogen, TAG, and protein in diapausing and recovering *Canton S* flies.

Whole body glucose concentrations are significantly increased (1.2–1.7 fold) during the course of the diapause compared to controls kept at non-diapause conditions, with a maximum at 2 weeks of diapause (Fig. 3C). These glucose levels drop significantly (to C1 levels) after recovery from 3 and 6 weeks of diapause (R1′–R2 in Fig. 3C). Newly eclosed flies (C0) display only 77% of the body glucose of non-diapausing C1 flies. Trehalose stores in whole bodies peaked after 3 weeks of diapause (4-fold higher than in C1 controls) and dropped to C1 control levels after 9 and 12 weeks (Fig. 3D). Flies that had recovered from diapause displayed trehalose levels similar to corresponding diapausing flies. The newly eclosed C0 flies displayed trehalose stores similar to C1 controls (Fig. 3D).

Stored glycogen and TAG, which are important during insect diapause [5], display different patterns of change in diapausing flies. Compared to C1 control glycogen levels are reduced by about 50% in flies after 1 week of diapause conditions (D1) and then increased with a maximum after 3 weeks (28% increase compared to C1), followed by a decrease 20% below levels in the C1 control (Fig. 3E). The newly eclosed C0 flies stored significantly less glycogen than C1 controls (Fig. 3E). Finally, we found that the TAG levels are elevated 2.2–4.8 fold throughout diapause with a peak at three weeks compared to C1 flies (Fig. 3F). Recovery from diapause does not significantly affect glycogen and TAG levels, but lipid stores are larger in recovered flies than in C1 controls (Fig. 3E,F). The 3–6h old C0 flies display significantly larger TAG stores than C1 controls (Fig. 3F).

Circulating proteins and amino acids may play a role in cryoprotection and cold-induced diapause (reviewed in [53], [58]). Thus, the hemolymph was shown to contain increased levels of amino acids/proteins in diapausing adult females of Colorado potato beetle [59] and larvae of European corn borer [60]. We observed a substantial decrease in circulating protein in hemolymph of 1-week old non-diapausing controls (C1) compared to 3–6 h old flies (C0). In diapausing flies (D1–D3) there is a significant increase of circulating protein compared to C1 flies (Fig. 3G). A return of circulating protein to C1 levels is seen in flies that were placed for 1 week (R1′) under normal conditions after 3 weeks of diapause, but the recovery after 6 weeks diapause leads to increased levels (Fig. 3G). Levels of whole body protein did not change over the 12 weeks of diapause (Fig. 3H). However, whole body protein is higher in the newly eclosed flies (C0) compared to C1 control flies (Fig. 3H).

In summary, we found that in *D. melanogaster* of the *Canton S* strain diapause conditions lead to increased levels of circulating carbohydrates and proteins, and these remain elevated after recovery from diapause. In addition diapause is accompanied by an increased accumulation of stored carbohydrates and TAG, which are also not depleted during the recovery periods tested here.

### Diapause-induced changes in gene expression related to insulin- and glucagon-like signaling and metabolism

Diapause in insects and the diapause-like dauer stage of *C. elegans* are the results of complex alterations of metabolism, energy stores, stress resistance and growth, and are likely to be under regulation by insulin/IGF signaling (IIS) [1], [2], [5], [14], [19], [36], [61]. In *D. melanogaster* one of the components of the IIS pathway, phosphoinositol-3-kinase, PI3K, was found to be important for diapause induction [20] and in a mosquito the insulin receptor, one of its ligands (insulin-like protein 1, ILP-1) as well as the forkhead transcription factor FOXO were found critical for reproductive diapause [19], [62]. We therefore used quantitative real time PCR (qPCR) to monitor expression of genes related to insulin signaling in diapausing flies. Three DILPs (DILP2, 3 and 5) are coexpressed in insulin producing cells (IPCs) of the brain, but are independently regulated at the transcriptional level [25], [40], DILP6 is primarily expressed by fat body cells [63], [64], but its role in adult physiology is just starting to be investigated [65]. Alterations in transcript levels of *dilps* does not necessarily tell us whether specific DILPs are actually released, but are useful read-outs of responses to changes in organismal physiology. For instance, in larvae starvation diminishes *dilp3* and *dilp5* transcripts, but has no effect on *dilp2* [40].

**Figure 4:**
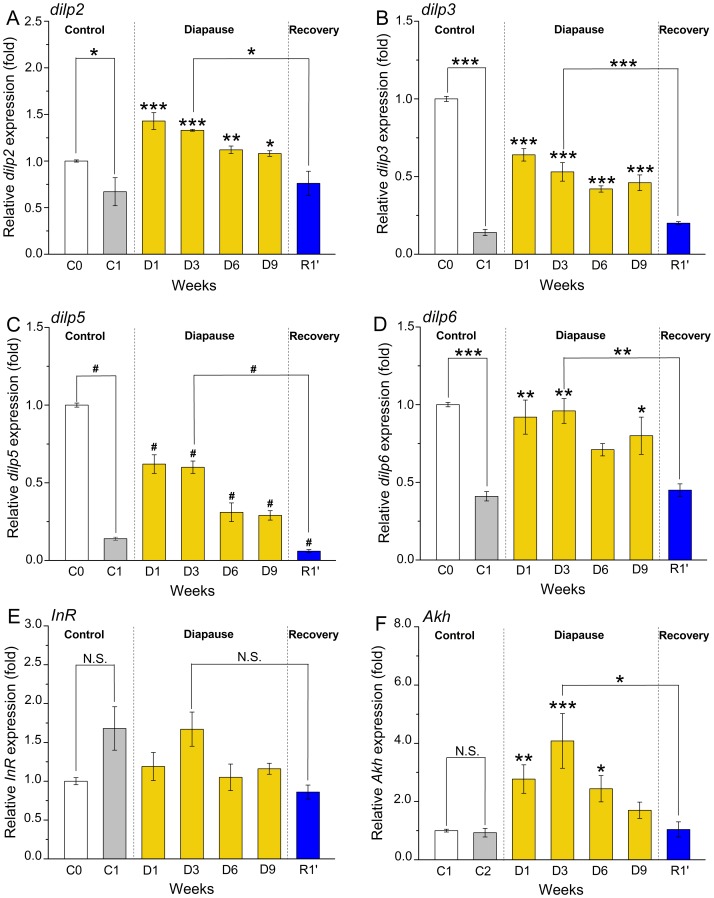
Altered gene expression in diapausing flies assessed by qPCR suggest endocrine diapause phenotypes. Relative steady state expression of genes encoding DILPs, InR and AKH in virgin female flies (*Canton S*), kept for 1–9 weeks under diapause conditions (11°C and 10L:14D), and for one week recovery (R1′) under non-diapause conditions (25°C and 12L:12D) after 3 weeks of diapause. Virgin flies kept for one week at normal conditions (C1) were used as comparison. The expression values were calculated with the 2^−ΔΔCt^ method relative to that of the 3–6-h old virgin flies (C0) in each assay. Data are presented as means ± S.E.M, *n* = 4 independent replicates with 10–15 flies in each replicate. Significance of differences from 1 week non-diapause control (1) is indicated as well between groups indicated by connectors, *p*<0.05, ** *p*<0.01, *** *p*<0.001, N.S. not significantly different (ANOVA followed with Tukey test) or alternatively ^#^ *p*<0.05, ^##^ *p*<0.01 or ^####^ *p*<0.01 (Kruskal–Wallis test followed by pairwise comparisons using Wilcoxon rank sum test). **A** Compared to 1-week old non-diapausing flies (C1) the *dilp2* expression increased significantly after one week of diapause (D1) and remained significantly higher over diapause (D3–D9). After one week of recovery from diapause (R1′) the *dilp2* expression decreased significantly back to the C1 level. Interestingly the recently hatched control flies (C0) display a significantly higher *dilp2* expression compared to 1-week non-diapausing flies (C1). **B** Compared to C1 controls *dilp3* increased after one week of diapause and remained higher during 9 weeks (D1–D9). After one week of recovery (R1′) from diapause *dilp3* decreased back to the control level (C1). The *dilp3* expression is significantly higher in recently eclosed flies (C0) than in one week normal controls (C1). **C** The *dilp5* expression showed a profile similar to that of *dilp3*. **D** The *dilp6* expression was significantly higher throughout diapause than in C1 controls, but with a slight decrease after 6 weeks (D6). After one week of recovery from diapause *dilp6* decreased significantly. Recently hatched flies (C0) display higher *dilp6* expression than the one week old ones (C1). **E** The insulin receptor (*InR*) transcript displayed no significant differences between the treatments. **F** The *Akh* mRNA increased drastically after one week in diapause (D1) and peaked after three weeks (D3). The transcript level returned to the one week control (C1) level after recovery from diapause. The *Akh* expression is not significantly different in recently eclosed flies (C0) and one week normal controls (C1).

All four investigated *dilps* (*dilp2, 3, 5 and 6*) display similar expression profiles in *Canton S* flies under the experimental conditions (Fig. 4A–D). Thus, in newly eclosed flies (C0) *dilp* mRNA levels are higher than in flies kept for 1 week at normal conditions (C1) (Fig. 4A–D). The largest differences are observed for *dilp3* and *dilp5* transcripts, where C1 flies show only 14% of the values seen in C0 flies (Fig. 4B–C). In flies subjected to diapause conditions, we observed by 2.2–4.5-fold elevated levels of all four *dilps* already after one week of diapause (D1) compared to non-diapausing flies (C1) (Fig. 4A–D). High relative expressions were also registered in 3-week diapausing flies (D3). After 6–9 weeks of diapause (D6–D9) *dilp* levels decrease, but are still higher than in the C1 non-diapausing flies (Fig. 4A–D). Flies that had recovered for one week from three weeks of diapause (R1′) display a decrease in all four *dilp* transcripts to the control (C1) levels (Fig. 4A–C). In contrast to alterations in expression of *dilps*, the insulin receptor, *InR* mRNA does not change significantly over 9 weeks of diapause or after recovery compared to non-diapausing flies (C1) and is similar to that of the newly eclosed flies (Fig. 4E).

Another peptide hormone involved in regulation of carbohydrate and lipid metabolism in *Drosophila* is glucagon-like adipokinetic hormone, AKH [51], [52], [54], [66]. We recorded a 4.4-fold increased *Akh* expression in flies after 3 weeks of diapause (D3) and it remains about twofold up-regulated up to 9 weeks (D9) in comparison to non-diapausing flies (C1) (Fig. 4F). Recovery for one week from three weeks of diapause (R1′) leads to a diminishment of *Akh* expression to the level observed in the control flies (C1) (Fig. 4F).

**Figure 5:**
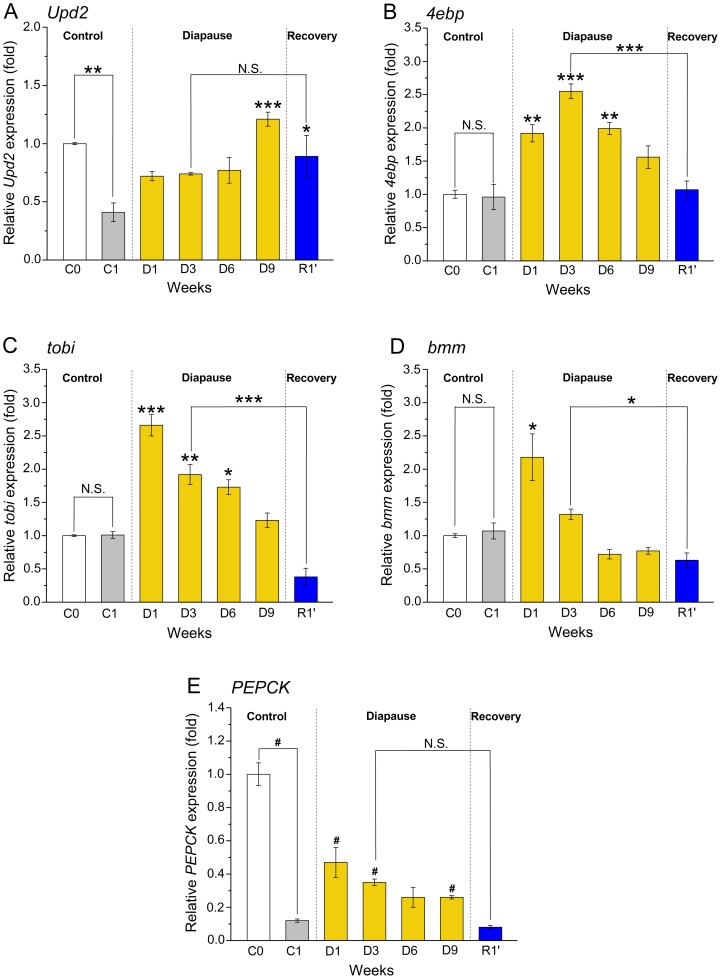
Changes in gene expression determined by qPCR suggest metabolic diapause phenotypes. Relative expression of genes encoding the cytokine Unpaired-2 (*Upd2*), the translational inhibitor 4EBP (*4ebp*), the α-1,4-glucosidase Target of brain insulin (*Tobi*), Brummer TAG lipase (*bmm*) and the lipid metabolism regulator *PEPCK* (phosphoenolpyruvate carboxykinase) in virgin female flies (*Canton S*), kept for 1–9 under diapause conditions (D1–D9) and for one week recovery (R1′) at normal conditions after 3 weeks of diapause, as well as in virgin flies 3–6 h after eclosion (C0) and one week old flies (C1) kept under non-diapausing conditions. Sampling and statistics are the same as in Fig. 4. **A** The *Upd2* expression in 9-week diapausing flies (D9) and after recovery (R1′) are significantly above the level in non-diapausing controls (C1). The *Upd2* expression was lower in one week controls (C1) than newly hatched ones (C0). **B** *4ebp* expression increases during diapause with a maximum at three weeks (D3) compared to C1 flies, but decreases back to the control (C1) level after recovery (R1′). **C** The *tobi* mRNA level in 1-week diapausing flies (D1) drastically exceeds that seen in 1-week non-diapausing flies (C1) and remains higher until 6 weeks of diapause (D6). After recovery (R1′) *tobi* expression is back to the control level (C1). **D** During diapause there is no significant change in *bmm* expression compared to the non-diapausing control (C1), with exception of 1-week diapause (D1), where the transcript level is increased. **E** The level of PEPCK mRNA is increased during diapause (D1, D3 and D9) compared to (C1) flies, and after recovery (R1′) it returns to the level of C1. The *bmm* expression is much higher in 3–6 h old flies (C0) than in one week old control flies (C1).

A recently identified fat body-derived factor is the cytokine Upd2 (unpaired-2), which displays leptin-like properties in *Drosophila* [67]. The *Upd2* displays significantly lower expression in 1-week old control flies (C1) than in recently eclosed ones (C0) (Fig. 5A). In diapausing flies there is a significant increase in *Upd2* transcript level only after 9 weeks (D9) (Fig. 5A). In flies that recovered from diapause (R1′) relative *Upd2* expression is not changed significantly from the diapausing flies (D3) and remains higher than that in the non-diapausing flies (C1) (Fig. 5A).

We next measured transcripts of genes that are either considered targets of insulin signaling or genes, which are involved in regulation of protein, carbohydrate and lipid homeostasis in response to nutrient levels in the fly. First we monitored the expression of a target of insulin/FOXO, the eukaryotic initiation factor 4 binding protein (4EBP; also known as Thor) that is an inhibitor of translation and protein synthesis. Up-regulation *4ebp* indicates down-regulation of IIS and resulting dFOXO activation [21], [68], [69]. We monitored a 2.0–2.7-fold increased *4ebp* expression throughout diapause compared to non-diapausing flies (C1), with a peak after 3 weeks (D3) (Fig. 5B). Flies that had recovered for 1 week from 3 weeks of diapause (R1′) display the same levels of *4ebp* as the controls (C1) Fig. 5B). We did not find any differences in *4ebp* expression between newly eclosed flies (C0) and those kept for 1 week at normal conditions (C1) (Fig. 5B).

Another target of DILP signaling is the gene *target of brain insulin (tobi)*, that encodes an α-glucosidase involved in hydrolysis of glycogen [70]. The *tobi* mRNA is drastically increased in flies after one week of diapause (D1) compared to non-diapausing controls (C1) (Fig. 5C). After 1 week of diapause *tobi* expression decreases slightly, but remains significantly above the control level (C1) for 6 weeks (Fig. 5C). In flies that recovered from diapause (R1′) *tobi* levels decreases to the control level (C1) (Fig. 5C). The 3–6-h old flies (C0) show the same level of *tobi* expression as the non-diapausing control flies (C1) (Fig. 5C).

The relative expression of the gene *brummer (bmm)*, encoding Brummer TAG lipase, a regulator of fat stores [71], does not differ between C1 and C0 flies (Fig. 5D) similar to the relative *4ebp* and *tobi* expressions (Fig. 5B–C). The only significant increased *bmm* expression (two-fold) compared to C1 flies is seen after 1 week of diapause (D1) (Fig. 5D). However, the *bmm* level decreases during the later stages of diapause (D3–D9) as well as during recovery (R1′) (Fig. 5D). Another regulator of lipid stores is the phosphoenolpyruvate carboxykinase PEPCK, which is can increase TAG stores via the glyceroneogenesis pathway [72]. We found that *PEPCK* expression is drastically higher in newly eclosed 3–6-h old flies (C0) than in control flies kept for 1 week at normal conditions (C1) (Fig. 5E). In diapausing flies (D1–D9) *PEPCK* expression levels significantly increase (2.2–3.9-fold) compared with to non-diapausing flies (C1), but return to levels in control flies (C1) after recovery from diapause (R1′) (Fig. 5E).

Our data suggest that the effect of diapause on gene expression in *D. melanogaster* is not a simple decrease of IIS, as suggested in studies of the mosquito *Culex pipiens* [19], [62]. In diapausing flies we observed simultaneous up-regulation of four of the *dilp*s and *Akh*, accompanied by increased expression of *tobi*, *4ebp*, *PEPCK* and *bmm*. However, it should be noted that the transcript levels of the *dilps* and *Akh* does not inform us about secretion of the peptides into the circulation, but the alterations in expression of the other genes are congruent with the increased levels of carbohydrates and lipids and are likely to reflect a shifted regulation of metabolic homeostasis at low food intake and decreased locomotor activity.

### Effects of diapause on the intestine

**Figure 6:**
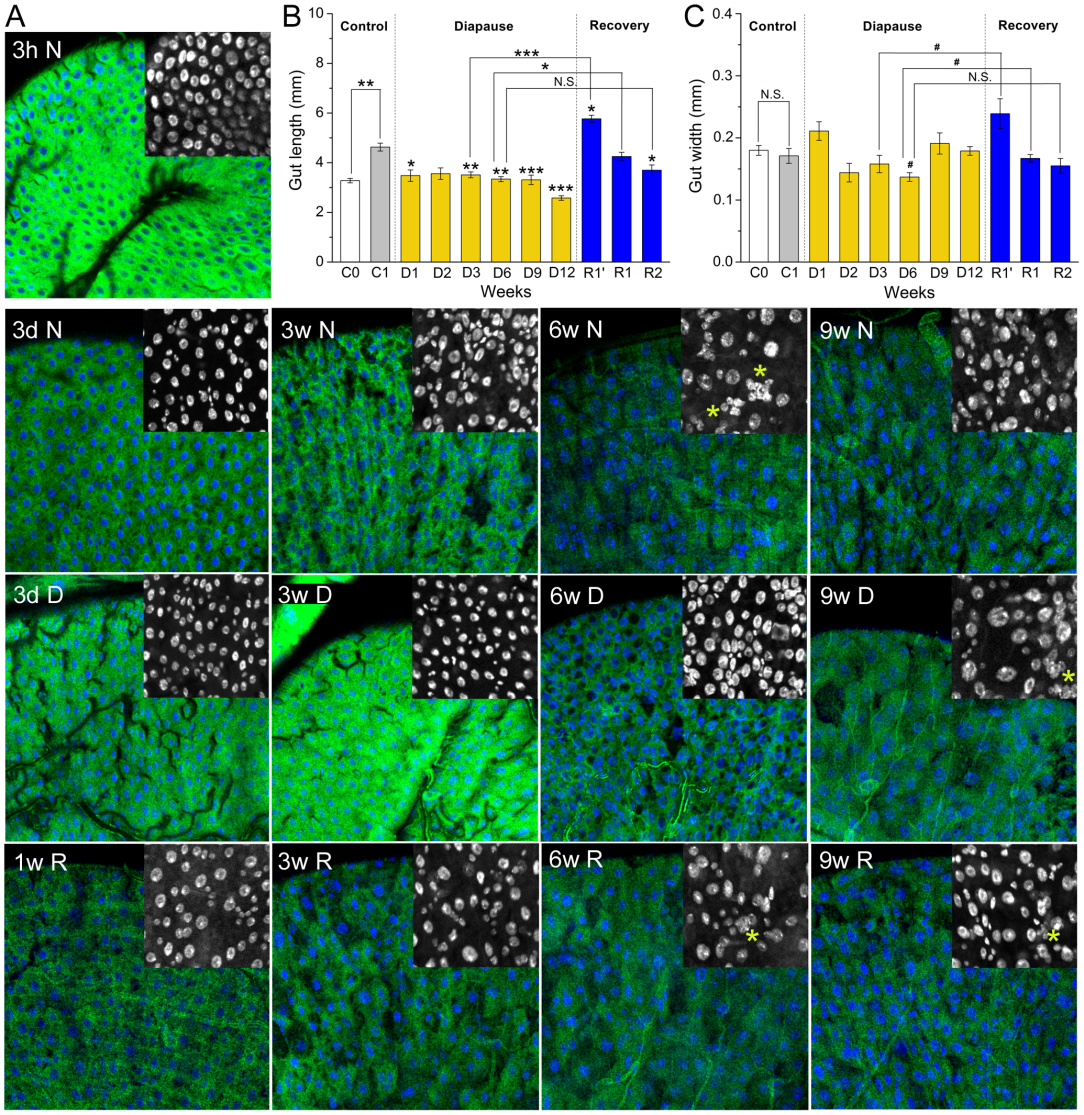
Effects of diapause conditions on gut-related structures in *Canton S* flies. **A** The intestinal epithelium appears to age more slowly during diapause. The epithelial cells (EC) were marked with NP1-Gal4 driven expression of GFP (green) in combination with nuclear staining with Hoechst 33342 (blue). The insets display enlarged view of nuclear staining. The flies tested were kept under control and diapause conditions as described earlier for 3 h (3 h N) or 3 days normal conditions (3 dN), 3–9 weeks normal conditions (3–9 w N), 3 days (3 d D) or 3–9 weeks diapause conditions (3–9 w D) and finally for 1–9 weeks recovery conditions after 6 weeks of diapause (1–9 w R). The age-associated changes in growth of EC size and disruption of the EC monolayer in the midgut are delayed by at least 3 weeks in diapausing flies. The yellow asterisks indicate small polyploid cells (sign of intestinal dysplasia). **B** The length of the midgut was measured during diapause (conditions as in Fig. 2A). The midgut is significantly longer in flies kept 1-week at normal conditions (C1), know to feed properly, than in 3–6 h old flies (C0). In diapausing flies (D1–D12) the midgut is shorter than in C1 controls and then becomes significantly longer after recovery from diapause (R1′, R1–2). Data are presented as means ± S.E.M, *n* = 6–9 randomly selected flies for each sample point. Significance of differences from the 1-week control (C1) is indicated, as well as between groups indicated by connectors, * *p*<0.05, ** *p*<0.01, *** *p*<0.001, N.S. not significantly different (ANOVA followed with Tukey test). **C** The width of the midgut did not change much during diapause, except a significant increase after 1-week recovery from diapause (R1′, R1). Data are presented as means ± S.E.M, *n* = 5–8 randomly selected flies for each sample point. Significance of differences from the 1-week control (C1) is indicated, as well as between groups indicated by connectors, ^#^ *p*<0.05 (Kruskal–Wallis test followed by pairwise comparisons using Wilcoxon rank sum test).

So far we have shown that reproductive diapause causes a reallocation of energy resources, slows down feeding and decreases body mass in *Canton S* flies. It is likely that diapause also reduces activity in the intestine. We found that the length of the midgut changes during diapause. The gut length in diapausing flies is significantly shorter than in one week-old control flies (C1), that feed normally (Fig. 6B), but similar to that of 3–6 h controls (C0). Flies that recovered for one week from 3 weeks of diapause (R1′) display drastically increased length of the midgut even exceeding that of one week control flies (Fig. 6B). The diameter of the midgut does not change during diapause, although a significant increase was seen after one week of recovery from 3 weeks in diapause (Fig. 6C). The morphological changes seen are possibly induced by lower food intake, known to result in decreased rate of proliferation of intestinal stem cells (ISCs) [48], [73]. The increase in length of the midgut at recovery from diapause may reflect a mechanism similar to that seen at refeeding after food deprivation, which triggers proliferation of ISCs and increased gut length [48], [74].

For a more detailed view of changes in the intestine we monitored the midgut in diapausing flies by driving GFP expression in cell membranes of enterocytes (ECs, gut epithelial cells) with the NP1-Gal4-driver [75] in combination with a nuclear staining with Hoechst dye (Fig. 6A). Control flies reared in non-diapausing conditions display the strongest NP1 expression during the first 3 hours of life. This signal gradually decreases within 24 hours and after 3 weeks the fly intestine displays a complete loss of NP1 signal in some ECs indicating cell death caused by aging processes and increased demand for EC turnover. However, in midguts of diapausing flies the decrease in NP1 expression during the first days of life was much slower and after 3 weeks of diapause it was even restored to levels comparable with 3 hours old flies. In addition, we observed loss of NP1 signal only after 6 weeks of diapause (Fig. 6A). In 3 weeks old control flies and later stages we also detected an increase in nuclear size in most of the ECs, whereas in diapausing flies this feature is visible only after 6 weeks and later. Another phenotype that is usually connected to gut aging is progression of intestinal dysplasia [73]. We detected clusters of small polyploid cells that accumulate at the basal membrane of the epithelium in 9 weeks old diapausing flies (and older), whereas in control flies those clusters are visible already after 6 weeks (Fig. 6A). Analyzing Hoechst staining of nuclei in midguts that had recovered from diapause for one or two weeks we detected an increased number of cells with small nuclei. These are likely nuclei of proliferating ISCs and their rapidly differentiating daughter cells [76], which give rise to a growth of gut epithelium that may correspond to the increase in midgut length and width seen after recovery (Fig. 6B).

### The innate immune system is activated by diapause

**Figure 7:**
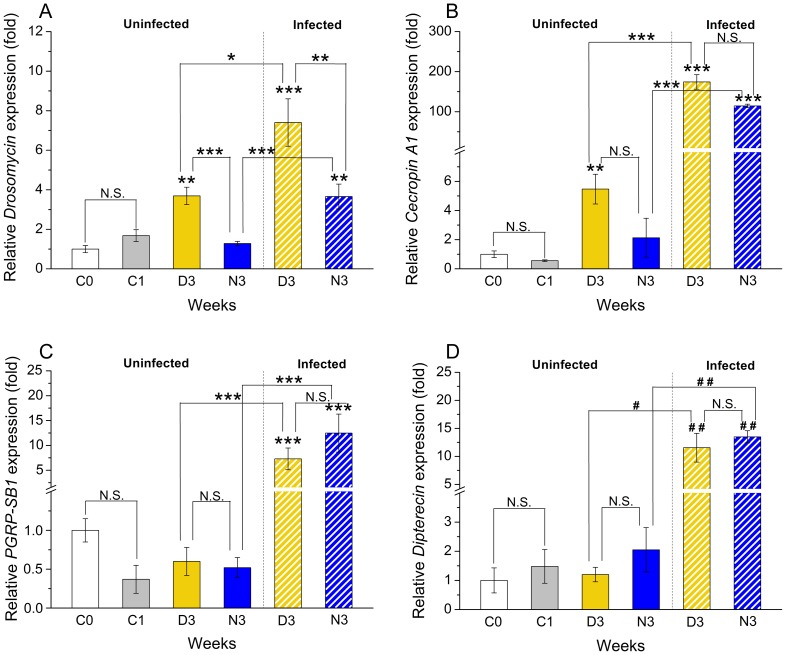
Selective effects of diapause conditions on expression of innate immune genes. The relative expression of four immune genes was determined in six groups of female *Canton S* flies: virgin 3–6 h old flies (C0), one week old uninfected and non-diapausing flies (C1), uninfected flies kept for 3 weeks either at 11°C and 10L:14D (diapause, D3) or at 25°C and 12L:12D, (normal conditions, N3) and infected flies (cross hatched bars) kept under diapause (D3) or non-diapause conditions (N3) for 3 weeks. Infected flies were injected with a suspension of *Micrococcus luteus* and *Escherichia coli* and kept for an additional 3 hours before freezing and RNA extraction. Data are presented as means ± S.E.M, *n* = 3–4 replicates with 10–15 flies in each. Significance compared to the newly hatched control (C1) which was set at one, or as indicated by connectors: * *p*<0.05, ** *p*<0.01, *** *p*<0.001, N.S. – not significant (ANOVA followed with Tukey test) or ^#^ *p*<0.05, ^##^ *p*<0.01 (Kruskal–Wallis test followed by pairwise comparisons using Wilcoxon rank sum test). **A** The *Drosomycin* expression was significantly upregulated in flies diapausing for 3 weeks (D3) in both infected and uninfected flies compared to non-diapausing 1-week old (C1) and 3-week old flies (N3). Infection further increased transcripts in both N3 and D3 flies. **B** *Cecropin A1* was significantly upregulated during diapause (D3) versus normal conditions (C1), but not N3, in uninfected flies only if comparing C1 to D3. Infection drastically increased transcripts in both diapausing and nondiapausing flies. **C** The *peptidoglycan recognition protein SB1* (*PGRP-SB1*) transcript level are not affected by diapause, only infection increased it. **D** *Diptericin* also increased only due to infection. For the investigated immune genes we did not find a differences in expression levels between 1-week old (C1) and newly eclosed (C0) flies. Similar results were obtained with flies that were reared on food supplemented with antibiotics (see Fig. S2 in File S1).

Innate immunity is likely to play a critical role during insect diapause and genes of the Toll/Imd pathways are known to display strong allelic variation in a North American cline of *D. melanogaster* that show variations in important life history traits [77]. We set out to analyze expression of a selection of key immune genes in diapausing flies. Both infected and uninfected flies were analyzed. Uninfected flies were kept for 3 weeks in diapause or non-diapause conditions, and one week of normal conditions. For infected specimens we injected flies previously kept for 3 weeks at 11°C and 10L:14D, and non-diapausing control flies of the same age, with a mix of Gram-negative (*Escherichia coli*) and Gram-positive (*Micrococcus luteus*) bacteria. Three hours after bacterial injection flies were sampled, along with non-infected flies, for analysis of gene expression. We examined the transcriptional activation of three antimicrobial peptide (AMP) genes and one peptidoglycan recognition protein (PGRP-SB1) gene regulated by the Toll and Imd (immune deficiency) pathways (Fig. 7).

We found a significant and strong activation of the *Drosomycin* gene after infection in diapausing flies compared to infected flies kept in normal conditions (Fig. 7A). Furthermore, compared to uninfected flies, we detected an increased transcriptional level of *Drosomycin* in naïve diapausing flies compared to non-diapausing flies (Fig. 7A). It can be noted that despite its constitutive upregulation in diapausing flies the *Drosomycin* transcript is also significantly elevated by microbial elicitors. Another Toll-regulated gene, *Cecropin A1*, showed a non-significant induction in uninfected three-week diapausing flies compared to three-week non-diapausing ones (Fig. 7B). However, this gene was significantly upregulated (9–8-fold) compared to one week-old non-diapausing flies (Fig. 7B). Infected flies displayed strong induction of *Cecropin A1* in both groups (Fig. 7B). The transcript levels for *Diptericin* and *PGRP-SB1* genes, which are regulated mostly by Imd pathway [78], [79], were significantly elevated after infection in both diapausing and non-diapausing flies (Fig. 7C, D). However, RNA levels were not significantly different when comparing diapausing and non-diapausing flies (Fig. 7C, D). Thus, in summary *Drosomycin*, and to a lesser extent *Cecropin A1*, are upregulated in diapausing flies in absence of obvious infection, possibly as part of an increased preparation for surviving harsh conditions. Furthermore we show that all four immune genes tested can be strongly induced in diapausing flies after bacterial infection. This increased expression was neither influenced by age nor some hidden infection. We tested flies kept on food supplemented with antibiotics and no effect was seen (Fig. S2 in File S1).

### Diapause induction in insulin deficient mutants

Insulin signaling plays a role not only in carbohydrate and lipid metabolism in insects, but may also be important in diapause induction and maintenance [5], [12], [14], [19], and we noted effects of diapause on transcript levels of *dilp2, 3, 5 and 6* and likely target genes of insulin signaling. Thus, we analyzed the effects of genetic knock-down of *dilp* expression on diapause induction and metabolic homeostasis. We utilized two mutant fly lines, a *dilp5* and a combined *dilp2-3* mutant, both in *w^1118^* background [21] to monitor effects of diapause-, conditions on ovary development, feeding, carbohydrate and lipid metabolism. Data from the mutant flies are shown in Tables 1 and 2 (and graphically in Fig. S3–6 in File S1) and data from *w^1118^* are displayed in Table 3. A comparison of the phenotypes at three weeks of diapause in the mutant and wild type strains (*Canton S* and *w^1118^*) is shown in Table 4, and a comparison of one-week-old non-diapausing flies (C1) of the four strains in Table 5.

**Table 1:** Effects of diapause and other conditions on *dilp2-3* mutant flies.

	Week	Hemolymph			Body				
		Protein (µg/µL)	Glucose (mM)	Trehalose (mM)	Glycogen (µg/mgwm)	Glucose (µg/mgwm)	Trehalose (µg/mgwm)	TAG (µg/mgwm)	Protein (µg/mgwm)
Control (C)	C0	6.96±0.23	13.9±0.6	0.12±0.02	3.95±0.26	5.97±0.34	1.63±0.37	0.45±0.06	43.5±0.8
	C1	3.94±0.10	9.97±0.21	0.11±0.01	15.2±0.4	7.48±0.07	1.02±0.07	0.37±0.02	45.8±0.5
	vs C0	***	^##^	N.S.	***	N.S.	N.S.	***	N.S.
Diapause (D)	D1	6.81±0.19***	16.8±0.6^#^	0.41±0.11*	10.3±0.3***	10.7±0.6***	3.64±0.48	1.21±0.08***	46.4±0.6
	D2	5.46±0.12*	16.3±0.8^#^	0.47±0.08**	18.2±1.1	12.2±0.3***	5.59±1.55	1.19±0.13**	38.9±1.3***
	D3	5.59±0.39*	19.3±2.7^##^	0.48±0.08**	18.1±0.9	12.8±0.3***	7.47±1.16***	1.17±0.19**	37.9±1.1***
	D6	5.49±0.45*	23.5±1.9^#^	0.42±0.03**	14.1±0.7	12.8±0.3***	5.75±1.22*	0.81±0.09**	36.4±0.5***
	D9	7.77±0.05***	31.5±0.9^#^	0.46±0.09*	11.8±1.1*	12.1±0.6***	2.08±0.29	0.86±0.11**	35.2±0.9***
	D12	7.13±0.10***	40.8±1.4^#^	0.43±0.09*	12.2±0.3	13.5±0.2***	2.55±0.73	0.87±0.16*	34.2±0.5***
Recovery (R)	R1′	5.26±0.15	20.5±0.4^#^	0.26±0.03	22.8±1.0***	11.0±0.3***	2.29±0.56	0.49±0.04	39.1±0.3**
	vs D3	N.S.	N.S.	N.S.	N.S.	N.S.	*	**	N.S.
	R1	5.40±0.17**	24.9±1.3^#^	0.30±0.08	17.2±0.4	10.9±0.2***	1.75±0.61	0.60±0.07	40.1±1.3*
	vs D6	N.S.	N.S.	N.S.	N.S.	N.S.	N.S.	N.S.	N.S.
	R2	6.21±0.25***	22.0±1.2^#^	0.20±0.03	16.3±0.4	10.2±0.3**	1.17±0.04	0.79±0.09**	38.5±0.4***
	vs D6	N.S.	N.S.	N.S.	N.S.	**	N.S.	N.S.	N.S.

Macronutrient composition in hemolymph and body of flies kept for 1–12 weeks at 11°C and 10L:14D, light/dark (D1–D12) and after recovery for 1 week after 3 weeks of diapause (R1′), or 1 (R1) or 2 weeks (R2) after 6 weeks of diapause. Virgin flies, kept for 1 week at non-diapausing conditions (C1) and recently hatched 3–6 h old flies (C0) were used as a controls. Data are presented as means ± S.E.M, *n* = 3–5 independent replicates with 10–15 flies in every replicate. Significantly different either from the control (C1) with *p<0.05, ** *p*<0.01, *** *p*<0.001 or from the indicated group as assessed by ANOVA, followed by Tukey test or alternatively with ^#^ *p*<0.05, ^##^ *p*<0.01, ^###^ *p*<0.001 as assessed by Kruskal-Wallis test followed with Wilcoxon pairwise comparison. N.S. – values are not significantly different. Vs, versus.

**Table 2:** Effects of diapause and other conditions on *dilp5* mutant flies.

	Week	Hemolymph			Body				
		Protein (µg/µL)	Glucose (mM)	Trehalose (mM)	Glycogen (µg/mgwm)	Glucose (µg/mgwm)	Trehalose (µg/mgwm)	TAG (µg/mgwm)	Protein (µg/mgwm)
Control	C0	5.81±0.15	6.97±0.73	0.10±0.01	4.06±0.28	5.31±0.24	1.45±0.05	0.36±0.01	55.6±0.3
	C1	3.93±0.18	9.21±0.23	0.10±0.02	15.6±0.5	7.33±0.17	1.21±0.07	0.29±0.01	43.0±0.8
	vs C0	**	N.S.	N.S.	***	**	N.S.	^##^	***
Diapause (D)	D1	6.09±0.28***	10.7±0.7	0.16±0.02	10.3±0.4***	9.08±0.43*	1.74±0.27	0.67±0.06^#^	50.6±0.8***
	D2	5.17±0.38	11.2±0.5	0.19±0.04	21.3±0.9***	10.2±0.2***	1.86±0.34	0.54±0.10^#^	47.9±0.6*
	D3	5.40±0.07*	14.2±0.2**	0.21±0.03	21.5±0.2***	10.9±0.1***	2.20±0.42	0.62±0.01^#^	39.0±1.1
	D6	4.45±0.22	12.5±0.4	0.12±0.01	14.7±0.2	9.23±0.15*	2.05±0.42	0.50±0.02^#^	40.6±1.1
	D9	4.39±0.38	16.7±1.1***	0.11±0.03	11.7±0.1**	9.54±0.65**	2.05±0.26	0.52±0.07^#^	37.3±0.4*
	D12	4.87±0.43	18.7±0.9***	0.15±0.01	12.2±1.2*	10.1±0.3***	1.62±0.36	0.99±0.10^#^	35.4±0.1***
Recovery (R)	R1′	3.24±0.11	13.8±0.6**	0.13±0.02	18.9±0.9*	9.47±0.12**	1.02±0.05	0.53±0.07	38.6±1.2
	vs D3	***	N.S.	N.S.	N.S.	N.S.	N.S.	N.S.	N.S.
	R1	4.04±0.50	17.3±0.8***	0.12±0.03	17.3±0.5	9.49±0.19**	0.89±0.17	0.84±0.23^#^	35.5±1.0***
	vs D6	N.S.	**	N.S.	N.S.	N.S.	N.S.	N.S.	*
	R2	4.54±0.15	15.9±1.1***	0.11±0.04	21.2±0.2**	8.69±0.05	0.81±0.10	0.61±0.06^#^	39.5±0.6
	vs D6	N.S.	N.S.	N.S.	***	N.S.	N.S.	N.S.	N.S.

Macronutrient composition in hemolymph and body of flies kept for 1–12 weeks at 11°C and 10L:14D, light/dark (D1–D12) and after recovery for 1 week after 3 weeks of diapause (R1′), or 1 (R1) or 2 weeks (R2) after 6 weeks of diapause. Virgin flies, kept for 1 week at non-diapausing conditions (C1) and recently hatched 3–6 h old flies (C0) were used as a controls. Data are presented as means ± S.E.M, *n* = 3–5 independent replicates with 10–15 flies in every replicate. Significantly different either from the control (C1) with *p<0.05, ** *p*<0.01, *** *p*<0.001 or from the indicated group as assessed by ANOVA, followed by Tukey test or alternatively with ^#^ *p*<0.05, ^##^ *p*<0.01, ^###^ *p*<0.001 as assessed by Kruskal-Wallis test followed with Wilcoxon pairwise comparison. N.S. – values are not significantly different. Vs, versus.

**Table 3:** Effects of diapause and other conditions on *w^1118^* flies.

	Week	Hemolymph			Body				
		Protein (µg/µL)	Glucose (mM)	Trehalose (mM)	Glycogen (µg/mgwm)	Glucose (µg/mgwm)	Trehalose (µg/mgwm)	TAG (µg/mgwm)	Protein (µg/mgwm)
Control (C)	C0	6.36±0.40	12.3±1.0	0.21±0.02	5.39±0.19	4.74±0.14	2.94±0.10	0.67±0.03	28.7±0.4
	C1	3.87±0.10	10.2±0.5	0.10±0.01	12.7±0.4	7.11±0.13	1.05±0.11	0.33±0.02	39.6±0.8
	vs C0	***	N.S.	^#^	***	***	^##^	***	***
Diapause (D)	D1	5.43±0.13*	15.3±0.6	0.61±0.05^#^	9.45±0.76**	7.26±0.27	4.42±0.10^##^	1.25±0.05***	31.3±0.8***
	D2	5.10±0.34	17.2±2.2	0.70±0.10^#^	16.7±0.2**	11.2±0.6***	5.38±0.40^##^	1.40±0.12***	30.0±0.5***
	D3	6.30±0.16***	18.2±0.5**	0.46±0.11	16.2±0.5**	12.4±0.7***	6.19±0.38^##^	1.86±1.21***	24.8±0.9***
	D6	3.79±0.17	16.0±0.4	0.35±0.12^#^	14.4±0.8	11.2±0.2***	4.78±0.53^##^	1.37±0.02***	25.9±0.4***
	D9	7.00±0.43***	23.1±2.0***	0.21±0.06	9.54±0.15*	12.4±0.2***	1.58±0.12^#^	1.19±0.07***	21.7±0.1***
	D12	6.07±0.32***	20.3±2.1**	0.21±0.08	8.55±0.12***	13.0±0.1***	2.04±0.15^#^	1.03±0.04***	21.6±0.4***
Recovery (R)	R1′	6.62±0.34***	23.2±0.7***	0.15±0.02	14.2±0.5	9.54±0.27***	2.86±0.74^#^	1.08±0.03***	22.0±0.1***
	vs D3	N.S.	N.S.	N.S.	N.S.	***	^##^	**	N.S.
	R1	5.80±0.18***	23.0±0.3***	0.15±0.03	15.2±0.5	10.2±0.2***	1.48±0.13	1.15±0.11***	21.2±0.2***
	vs D6	**	N.S.	N.S.	N.S.	N.S.	^##^	N.S.	***
	R2	6.33±0.45**	23.8±1.8***	0.14±0.03	13.2±0.3	9.62±0.30***	1.37±0.31	1.06±0.04***	19.8±0.4***
	vs D6	***	*	N.S.	N.S.	N.S.	^#^	N.S.	***

Macronutrient composition in hemolymph and body of flies kept for 1–12 weeks at 11°C and 10L:14D, light/dark (D1–D12) and recovery (R) from diapause for 1 and 1–2 weeks at 25°C and 12L:12D after 3 weeks (R1′) or 6 weeks (R1 and R2) of diapause. Virgin flies, kept for 1 week at non-diapausing conditions (C1) and recently hatched 3–6 h old flies (C0) were used as a controls. Data are presented as means ± S.E.M, *n* = 3–5 independent replicates with 10–15 flies in every replicate. Significantly different either from the control (C1) with *p<0.05, ** *p*<0.01, *** *p*<0.001 or from the indicated group as assessed by ANOVA, followed by Tukey test or alternatively with ^#^ *p*<0.05, ^##^ *p*<0.01, ^###^ *p*<0.001 as assessed by Kruskal-Wallis test followed with Wilcoxon pairwise comparison. N.S. – values are not significantly different. Vs, versus.

**Table 4:** Comparison of metabolic phenotypes induced by three weeks of diapause in four strains of *D. melanogaster*: *Canton S*, *dilp5* and *dilp2-3* mutants and *w^1118^*.

Parameter	*w^1118^*	*dilp5*	*dilp2-3*
Food intake (ng/fly/6 h)	N.S.	N.S.	N.S.
Fly mass (mg)	+7%*	N.S.	−3%*
Glucose in hemolymph (mM)	+21%**	N.S.	N.S.
Trehalose in hemolymph (mM)	N.S.	N.S.	N.S.
Protein in hemolymph (µg/µL)	+14%**	N.S.	N.S.
Glucose in body (µg/mgwm)	N.S.	−15%***	N.S.
Trehalose in body (µg/mgwm)	+36%*	−52%*	+64%*
Glycogen amount (µg/mgwm)	N.S.	+25%***	N.S.
TAG content (µg/mgwm)	N.S.	−62%**	N.S.
Protein in body (µg/mgwm)	−48%***	−18%***	−20%***

Data are obtained from 3–6 h old virgin flies exposed to three weeks of diapause conditions (11°C and 10L:14D). This time point was chosen since the strongest effect on ovarian development was seen. The table shows significant differences to the wild type *Canton S* (*p<0.05, **p<0.01, ***p<0.001 as assessed by by unpaired Students' *t*-test. N.S. – values are not significantly different).

**Table 5:** Comparison of metabolic phenotypes in four strains of *D. melanogaster*: *Canton S*, *dilp5* and *dilp2-3* mutants and *w^1118^* kept under normal conditions.

Parameter	*w^1118^*	*dilp5*	*dilp2-3*
Food intake (ng/fly/6 h)	+86%*	+43%*	+120%***
Fly mass (mg)	+6%***	−7%***	−14%***
Glucose in hemolymph (mM)	+42%***	+29%***	+39%***
Trehalose in hemolymph (mM)	N.S.	N.S.	N.S.
Protein in hemolymph (µg/µL)	+29%***	+31%**	+32%**
Glucose in body (µg/mgwm)	−15%***	−12%***	−10%***
Trehalose in body (µg/mgwm)	N.S.	N.S.	N.S.
Glycogen amount (µg/mgwm)	−5%*	+16%^##^	+13%**
TAG content (µg/mgwm)	N.S.	−15%*	N.S.
Protein in body (µg/mgwm)	−23%***	−16%***	−11%**

The parameters were investigated in 3–6 h old flies *D. melanogaster*, kept for 1 week (7 days) at 25°C and normal photoperiod 12L:12D, light/dark, which correspond to the standard (normal) conditions in most studies with *D. melanogaster*. Significantly different from the wild line *Canton S* with *p<0.05, **p<0.01, ***p<0.001 as assessed by unpaired Students' *t*-test or ^##^ *p*>0.01 as assessed by Mann-Whitney-Wilcoxon rank sum test. N.S. – values are not significantly different.

We analyzed ovarian development in the *dilp2-3* and *dilp5* mutant flies under the same experimental conditions as used for *Canton S*. Importantly, in both *dilp5* and *dilp2*-*3* mutant flies there is normal vitellogenesis in virgin flies, kept for 1 week at non-diapausing conditions (C1), and in the newly eclosed flies (C0) the ovaries are fully previtellogenic (Fig. S3A–B in File S1). Both the *dilp2-3* and *dilp5* mutant flies differed significantly from wild type flies in ovary development during diapause. These mutant strains displayed decreased incidence of yolk accumulation and retarded ovarian development over 9–12 weeks of diapause conditions, and a slower recovery from 3 or 6 weeks of diapause (Fig. S3A–D in File S1). A comparison of yolk accumulation incidence between the different strains is shown in Fig. S3E in File S1. Thus, the ovarian development is more retarded in *dilp*-deficient flies than in *Canton S* and *w^1118^* flies (see next section) under the same experimental conditions, suggesting a more prominent diapause induction with diminished DILP signaling.

We next monitored food intake, body weight and carbohydrate and lipid metabolism in the two mutant strains. The food intake in *dilp5* and *dilp2-3* mutants, under the experimental conditions used for *Canton S* flies, displays a pattern similar to that we found for *Canton S* (Fig. 2A): very little food ingestion in newly eclosed (C0) and diapausing flies (D1–D12) compared to non-diapausing (C1) and recovered (R1′, R1–R2) ones (Fig. S4A–B in File S1). However, compared to *Canton S*, food intake in the two mutant lines was much higher in one-week control (C1) flies (120% more in *dilp2-3* mutants; in Figure S4A, B, in File S1, Table 5), whereas under diapause conditions there are no significant differences between the strains (Table 4; Fig. S4A–B in File S1).

Despite the increased feeding in the one week old non-diapausing (C1) *dilp* mutant flies, they had a significantly lower (7 and 14%, respectively) body mass than corresponding *Canton S* flies (Fig. S4C–D in File S1, Table 4B) and this difference is maintained in *dilp2-3* at 3-week diapause conditions (D3) (Fig. S4C–D in File S1, Table 4). Generally, the changes in body mass in diapausing mutant flies followed the same time course as *Canton S* flies (Fig. S4C,D in File S1; Table 4B).

Next we monitored changes in carbohydrate, protein and lipid levels under experimental conditions in the two *dilp*-deficient strains and compared them with *Canton S*. Circulating glucose levels are significantly higher in one week old controls (C1) in the two mutant strains than in *Canton S* (Table 1, 2, 4B; Fig. S5A,B in File S1). In both mutant strains the glucose increases over the 12 weeks of diapause. Significantly, in the *dilp2-3* mutants glucose reaches a quadrupled level after 12 weeks, compared to C1 flies (Table 2; Fig. S5B in File S1). These *dilp2-3* mutant flies actually display higher levels of circulating glucose at all tested conditions. After recovery from diapause glucose is not restored to control levels in the mutants or *Canton S* flies (Fig. 3A; Fig. S5A, B in File S1). Our data suggest that *dilp5* and especially *dilp*2-3 deficient flies are less efficient in regulation of blood glucose during diapause. The changes in circulating trehalose are similar in diapausing and non-diapausing *Canton S* flies and *dilp5* mutants, but absolute levels are higher in *dilp2-3* mutants and in the latter trehalose remained high throughout diapause (Table 1,2, 4, 5; Fig. S5C, D in File S1). Trehalose levels did not recover to C1 levels in the mutants or *Canton S*.

The stored (whole body) glucose levels are significantly lower in the *dilp* mutants than in *Canton S* during normal conditions (Table 5), but during diapause conditions *dilp5* files store less glucose than *Canton S* (Table 4). Similar to *Canton S* (Fig. 3C) the two mutants display increased body glucose stores during all time points of diapause (D1–D12), but these are not restored to control levels (C1) after recovery (Table 1–2; Fig. S5E–F in File S1). Whole body trehalose levels displayed different diapause profiles in the mutants compared to *Canton S* (Table 1,2; Fig. S5G, H in File S1). In *Canton S* flies stored trehalose peaks at 3 weeks diapause (twice the levels of C1 controls) and decreases continuously until 12 weeks (Fig. 3D), whereas in the *dilp*5 mutants trehalose levels are close to control levels throughout diapause (Table 1; Fig. S5G in File S1). Trehalose in the *dilp2,3* mutants rise to levels about seven times of the C1 controls at 3 weeks diapause and then drop similar to the wild type flies (Table 2; Fig. S5H in File S1). Finally, we did not see differences in body trehalose concentrations in the mutants and *Canton S* under non-diapausing conditions (Table 5).

There are no major differences in glycogen profiles in insulin deficient and *Canton S* flies during diapause (Table 1, 2, 4; Fig. S6A, B in File S1), except that *dilp5* mutants accumulate more glycogen after 3 weeks of diapause (Table 4). All lines demonstrate glycogen accumulation with a maximum at 2–3 weeks of diapause (D2–D3), and then a gradual decrease. After three weeks of diapause the glycogen level was significantly higher in *dilp5* mutants than the other strains (Table 4). Also, after 1 week of recovery from 3 weeks diapause *dilp2-3* and *dilp5* mutants displayed increased glycogen stores compared to C1 flies (Table 1 and 2; Fig. S6A, B in File S1), which was not seen in *Canton S* flies. Furthermore, the 1 week non-diapausing *dilp* mutants stored significantly more glycogen (Table 4B). The TAG contents increase in both diapausing *dilp2-3* mutants and *Canton S* flies, although the temporal profiles are slightly different; the wild type flies display peak levels at 3 weeks, whereas the *dilp2,3* mutants have more constantly elevated levels (Table 2; Fig. S6D in File S1). Compared to *Canton S* and *dilp2-3* mutants the *dilp5* mutants display lower levels of TAG in both controls (C1) and in diapausing (D3) flies (Table 1, 4, 5; Fig. S6C in File S1), but during diapause lipid stores increased also in *Dilp5* mutants compared to C1 controls (Table 1, Fig. S6C in File S1).

In comparison with *Canton S* flies the two mutants displayed approximately 30% higher protein levels in hemolymph under non-diapausing conditions (C1) (Table 5), whereas at 3 weeks of diapause no difference was seen between strains (Table 4). In the mutants circulating protein levels increase during diapause similar to wild type flies (Fig. 3G), although with a slightly larger increase in *dilp2-3* mutants than in *dilp5* (Table 1, 2; Fig. S6E–F in File S1). Whole body protein levels are significantly lower in the *dilp* mutants than in *Canton S* in non-diapausing controls (C1) as well as in 3-week diapausing flies (Table 4, 5). The changes in whole body protein during diapause are not significant in *Canton S* (Fig. 3H), whereas a steady decrease is seen in *dilp2-3* mutants (Table 2; Fig. S6H in File S1) and an initial increase followed by a decrease is noted in *dilp5* mutants (Table 1; Fig. S6G in File S1).

In conclusion, a lack of both *dilp2* and *dilp3* in flies exposed to diapause conditions results in a more drastic increment of circulating glucose and stored trehalose, but a moderate decrease of body protein. On the other hand flies with *dilp5* knockdown do not display substantial differences in carbohydrates, lipids or proteins under diapause conditions compared to the *Canton S* strain.

### Diapause induction in the *w^1118^* strain

The diapause phenotype of the *w^1118^* mutant strain of *D. melanogaster* was tested here since it is commonly employed as a control fly, due to the extensive use of its white eye color as a genetic marker [80], [81]. Furthermore, our *dilp* mutant flies are in *w^1118^* background [21]. Since the protein encoded by the *white* gene of *Drosophila* is a transmembrane ABC transporter involved in the uptake of guanine and tryptophan it has effects not only on the red and brown eye pigmentation, and thus light sensitivity, but also on biogenic amine levels in the nervous system [82], [83], [84], [85]. Thus, levels of histamine, dopamine and serotonin are drastically lowered in the brain of the *w^1118^* mutant, and male courtship behavior is altered [82], [83], [86]. Apart from this, the altered levels of biogenic amines may possibly affect feeding behavior and the function of the biological clock circuits (see [87], [88], [89]), both of which could play roles in diapause.

We assayed *w^1118^* flies exposed to the experimental conditions used for the other fly strains for ovary development, feeding, body mass and levels of carbohydrates, lipids and protein. The ovarian development clearly shows that low temperature and short days have a substantially weaker effect in *w^1118^* flies with at most about 40% incidence of yolk accumulation after 3 weeks diapause, compared to less than 10% in *Canton S* (Table 3; Fig. S3E, Fig. S7A, B in File S1). Food intake and fly mass followed a similar profile during diapause in *w^1118^* and *Canton S* (Table 3; Fig. S7C, D in File S1), although in *w^1118^* the amount of food eaten was higher in 1 week non-diapausing controls (C1) and in recovering flies, compared to wild type flies (about 80% larger food intake; Table 5). The levels of circulating glucose, but not trehalose, is higher in diapausing and non-diapausing *w^1118^* than *Canton S* flies (Table 3–5; Fig. S7E, F in File S1) and glucose was 42% higher in one week non-diapausing (C1) *w^1118^* flies than in *Canton S* (Table 5). Especially circulating trehalose levels display a different profile in *w^1118^* flies with a strong peak at 2 weeks diapause that is twice as high as the 3 week peak for *Canton S* (Table 3, 4; Fig. S7F in File S1). The whole-body glucose profile is similar in diapausing *w^1118^* and *Canton S*, while body trehalose is somewhat higher in *w^1118^* (Table 3, 4; Fig. S7G, H in File S1). Also glycogen and TAG stores are similar in the two fly strains under the different conditions (Table 3–5; Fig. S7I, J in File S1). In contrast both hemolymph and body protein levels different in *w^1118^* than in *Canton S* flies both in controls and diapausing animals (Table 3–5; Fig. S7K, L). In the one week old non-diapausing *w^1118^* flies (C1) body stores of glucose, glycogen and protein were significantly lower than in *Canton S* (Table 5). In summary it appears that *w^1118^* flies are less prone to diapause under the conditions tested here and, interestingly, we found that these flies kept under non-diapausing conditions differ quite a bit from *Canton S* in rate of feeding and several aspects of their metabolism.

### Mortality is negligible during diapause

We recorded mortality of the flies of the four different strains during the course of 12 weeks of diapause. As seen in Fig. S8 in File S1, mortality is very low in all four strains and found below 20% until 9 weeks of diapause and then around 30% at 12 weeks. Our study did not include mortality data for flies kept under non-diapausing conditions. However, published data for *w^1118^* and the *dilp2-3* mutant flies kept under normal conditions show that they do not survive until 12 weeks, and at 9 weeks less than 20% of the flies are alive [21].

## Discussion

**Figure 8:**
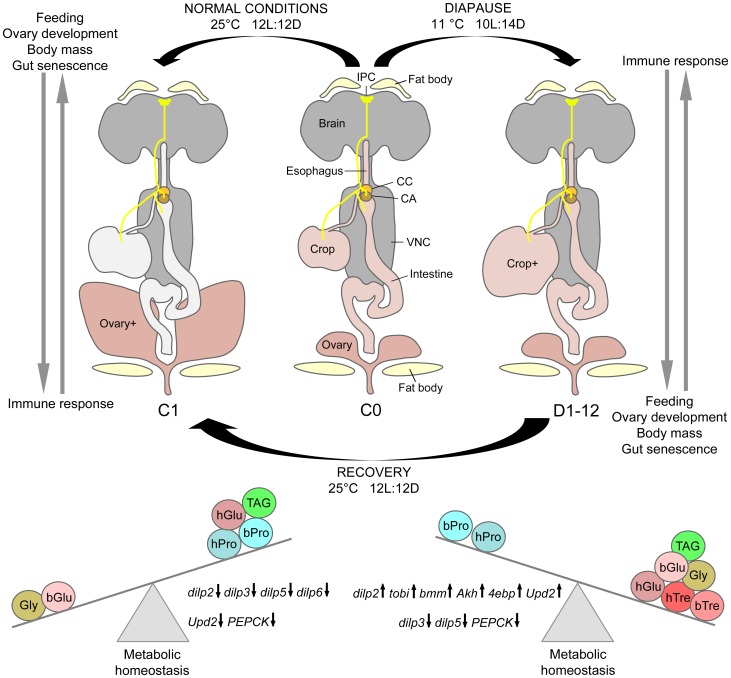
Reproductive diapause is an adaptive life-history trait in *D. melanogaster* induced by unfavourable environmental conditions. Under favourable conditions (25°C, 12L:14D) newly eclosed virgin flies (C0) with previtellogenic ovaries develop into a reproductive mature flies with vitellogenic ovaries (C1). However, if the initial C0 flies are exposed to unfavourable conditions (11°C, 10L:14D), they enter reproductive diapause (D) characterized by arrested ovarian development. Diapause in *D. melanogaster* is characterized by slowed maturation and aging, as indicated by previtellogenic ovaries and slowed aging of the gut epithelium. During diapause (D1–12) flies ingest very little food compared to control flies (C1). In diapausing flies there is an increase in hemolymph glucose (hGlu) and trehalose (hTre), body glucose (bGlu) and trehalose (bTre), as well as stored glycogen (Gly) and triacylglycerides (TAG), but neither in hemolymph (hPro) nor total body protein (bPro). Diapausing flies also display increased levels of some immune gene transcripts. The metabolic homeostasis is regulated by insulin producing cells (IPCs), endocrine cells of the corpora cardiaca (CC) and fat body cells. During diapause the expression profiles of several genes are altered: including *dilp*s (*Drosophila insulin-like peptides*), *Akh* (*adipokinetic hormone*), *tobi* (*target of brain insulin*), *bmm* (*Brummer lipase*), *PEPCK* (phosphoenolpyruvate carboxykinase), *Upd2* (*unpaired-2*) and *4ebp* (*eukaryotic initiation factor 4 binding protein*). Flies recover from diapause within a week of return to normal temperature and photoperiod (recovery), emphasizing that the dormancy serves during periods of unfavourable conditions. The outlines of the fly CNS, intestine and ovaries are redrawn from Toivonen and Partridge (2009) [116] with permission from L. Partridge.

Our study demonstrates that exposing newly emerged female *D. melanogaster* to low temperature and short photoperiod [13], [14], [41] triggers a set of dynamic alterations in physiology, gene expression and morphology that are congruent with entering a state of dormancy. More specifically, we suggest that the flies enter a reproductive diapause with retarded ovary development, altered carbohydrate, protein and lipid metabolism, changes in expression of a set of genes involved in metabolism and innate immunity, a slowed senescence of the intestine and a drastically reduced mortality. These findings are summarized in Fig. 8. In contrast to developmental diapause described in many insects (see [5]), the adult diapause of *D. melanogaster* is shallow and dynamic over time. While the diapausing fruitflies keep feeding at a low rate they are not simply consuming stored energy. Instead their homeostatic regulation of metabolism and energy allocation appears to be shifted to a different gear.

Previous studies of *D. melanogaster* have assayed ovary development, or monitored lifespan and resistance towards different stresses as a readout for diapause [10], [13], [14], [15], [16], [17], [41], [42]. It is clear that certain northern populations of *D. melanogaster* that are exposed to seasonal changes in environmental conditions enter an adaptive state referred to as diapause [9], [14], [90], [91], [92]. Although the *D. melanogaster* reproductive diapause is “shallow” [8], [12], [18] it displays similarities to that of insects with a programmed diapause (see [4], [5], [90], the facultative dauer state in *C. elegans* [1], [6], [61], or even hibernation in some mammals (see [93]). In order to establish *D. melanogaster* as a tractable model for adult diapause and associated adaptive life history traits, we investigated a more extended set of phenotypic alterations induced by conditions established earlier [13], [14], [41].

We found that the incidence of diapause differs between different laboratory strains of *D. melanogaster*. In the bulk of our experiments we employed *Canton S*, but found that the *w^1118^* strain, with a loss of function mutation in an ABC transporter [82], [83], [84], displays a shallower diapause as judged in several assays. The reason for this difference in *w^1118^* diapause is far from clear, but may relate to altered physiology and behavior due to the importance of this gene in monoamine biosynthesis [82]. Furthermore, two fly lines with loss of function mutations in genes encoding the insulin-like peptides *dilp2-3* and *dilp5*, appear to be more likely to diapause, as judged especially from ovary development, supporting the notion that insulin signaling plays an important role in diapause induction [5], [19], [20], [35], [90]. The importance of insulin signaling has also been suggested in developmental diapause and insects with programmed adult diapause [5], [12], [18], [19], [22], [35], [36]. Our study of *dilp* mutants reveals a difference to *Canton S* mainly in dynamics of ovary development, while differences in metabolic homeostasis under diapause conditions are subtler, and probably reflect that *D. melanogaster* simply shifts the homeostasis within a physiologically permissive range.

In this study we investigated flies of four different physiological states induced by environmental conditions. The first physiological state comprises the newly eclosed virgin flies (C0), which may represent a transition between the non-feeding pupal stage [63] and feeding adult fly [48]. These immature flies are the ones that can be induced to diapause by low temperature and short photoperiod or alternatively undergo a normal lifecycle when exposed to normal temperature and photoperiod. Surprisingly, newly eclosed flies have been little investigated before. We found that C0 flies feed very little the first 6–9 hours, and maintain high levels of circulating carbohydrates and proteins and store more lipids and proteins, but less glycogen than the control 1-week old flies (C1) which feed at a level almost ten times higher. The high levels of circulating and stored carbohydrates and lipids in newly eclosed C0 flies are accompanied by an elevated expression of *dilp2, 3, 5 and 6* compared to the fully feeding C1 flies. The other investigated genes, encoding coordinators of metabolic processes, as well as immune genes are at the same levels in C0 and C1 flies. Thus, newly eclosed flies with their previtellogenic ovaries are in a state of transition, switching metabolism from consumption of larval fat body to ingestion of nutrients.

The control virgin flies, kept under non-diapausing conditions for one week (C1), are in a state that corresponds to flies that are commonly used as controls in studies of metabolism and stress responses. Indeed, a C1 *Canton S* fly ingests around 50 ng of food over 6 h, which is very close to that found in mated flies of this strain [47]. It was noted that virgin flies feed less and have an increased intestinal transit than mated counterparts [94]. Although the C1 flies consume almost ten times more food than the newly eclosed (C0), they display a lower expression of *Upd2*, a functional analogue of mammalian leptin which is released from adipose cells after feeding [67]. The elevated *Upd2* expression in C0 compared to C1 flies correlates with higher *dilp2, 3 and 5* expression in C0 flies than in C1. Increased Upd2 activity triggers JAK/STAT signaling in GABAergic neurons that results in a disinhibition of the postsynaptic IPCs, and presumably increased DILP release [67].

When newly eclosed flies (C0) are subjected to 11°C and 12L:12D they enter diapause within a week. Thus flies kept under diapause conditions for a week (D1) display altered levels of carbohydrate, protein and lipid, changes in expression of a set of genes involved in regulation of metabolism and innate immunity. Over prolonged diapause these changes are accompanied with a slowed senescence of the intestine and a drastically reduced mortality compared to non-diapausing flies. For many of the parameters monitored over 12 weeks of diapause, we found that after three weeks (D3) the most drastic phenotypes could be recorded. Interestingly, we noted that the flies keep ingesting food, but at a very low rate throughout diapause. This means that metabolism is constantly fuelled, and in combination with lowered locomotor activity and arrested reproduction the fly is able to store nutrients similar to insects that prepare for developmental and programmed diapause [5], [53]. This altered resource allocation is reflected in a small decrease in body mass and abdomen size during diapause, but an increase in circulating glucose and trehalose, as well as stored carbohydrates and TAG over most of the diapause. Metabolic homeostasis and resource allocation in *Drosophila* is regulated by hormones produced by the brain and corpora cardiaca as well as factors produced by the fat body [30], [95]. The brain/corpora cardiaca hormones, DILPs and AKH, are functional homologs of vertebrate insulin and glucagon, respectively [24], [30], [40], [51], [54], [66], [70], [96]. Usually DILPs and AKH display antagonistic activities in metabolic regulation, but we found that during diapause the genes encoding both types of hormones are up-regulated. As a result we noted an increased expression of *tobi*, a gene responsive to both DILPs and AKH [70]. It seems that under unfavorable environmental conditions and with a reduced rate of feeding, the diapausing flies enter a new metabolic homeostasis with altered hormonal signaling. With both DILPs and AKH signaling at increased levels the diapausing flies can simultaneously accumulate nutritional stores (via DILP control) and maintain high level of circulating sugars (via AKH and DILP control). This resource allocation may ensure survival during diapause conditions with high circulating sugars as energy fuel and maybe cryoprotectant, with an energy reserve for survival during unfavorable conditions and for subsequent recovery from diapause. The alteration DILP and AKH signaling during diapause is reflected in gene expression in the fat body, a tissue with functions similar to vertebrate liver and adipose tissue [30], [95]. Thus in concert with increases of *dilp2*, *3* and *5* in the brain we noted an increase in *dilp6* RNA, presumably related to fat body expression. It is not clear how DILP6 affects adult physiology, although a possible feedback to IPCs of the brain has been suggested [65]. AKH in addition to its role in glycogenolysis [97], is also involved in lipid metabolism, independent of TAG Brummer lipase, BMM [66]. BMM, is a *Drosophila* ortholog of human adipocyte triglyceride lipase (ATGL) and is responsible for TAG mobilization mainly from fat body stores [71]. *Bmm* up-regulation, in 1-week diapausing flies may be related to the elimination of residual larval fat body droplets, found abundantly in flies at this stage. The alterations of *Akh* and *bmm* seems to have little impact on TAG stores in diapausing flies, maybe because of a compensation by the simultaneous increment of *PEPCK* expression. Both mammals and flies *PEPCK* has a role in gluconeogenesis as well as glycerogenesis [98]. Thus, like in mammals [99], there is a well-regulated balance between lipid synthesis (lipogenesis) and lipid mobilization (lipolysis), which adjusts lipid storage and ensures energy homeostasis under adverse environmental conditions, such as those during diapause in *Drosophila*.

We found an elevated transcription of *4ebp* in diapausing flies, which may indicate a peripheral activation of FOXO and reduced IIS [21]. However, *4ebp* is also known to be under a control of the TOR-signaling pathway [22], [32]. This may account for the retarded ovary development in diapausing flies, since it has been shown that in *Drosophila* 4E-BP activity is critical for survival under dietary restriction and environmental stress [68]. In addition *Thor*, a member of the 4E-BP protein family, is involved in induction of immune responses in *Drosophila* and other organisms [100]. Indeed, we found that the *Drosophila* diapause phenotype includes an upregulated expression of the antimicrobial peptide *Drosomycin*.

A diapausing fly is potentially more exposed to bacterial and fungal infection due to its more sessile life and is therefore expected to require an efficient defense. We detected a constitutive upregulation of two of the four tested immune genes in non-infected diapausing flies. One of these genes, *Drosomycin*, although predominantly regulated by the Toll pathway, receives additional input from insulin signaling [101], [102]. *Cecropin A1*, which is influenced by both pathways is upregulated compared to one-week non-diapausing flies, and two genes in the Imd pathway *PGRP-SB1* and *Diptericin*, did not display altered expression in non-infected diapausing flies compared to flies kept under normal conditions. However, all the immune genes were upregulated in infected animals, both in diapausing and non-diapausing flies. The increased expression of *Drosomycin* in diapausing compared to non-diapausing flies could be due to altered IIS. This could be accomplished by activation via a FOXO binding motif in the promoter of the *Drosomycin* gene [102]. Our findings, thus, suggest that *Drosomycin* is up-regulated during diapause in the absence of infection, possibly as a proactive response. We also show for the first time in *Drosophila* that the innate immune system is inducible during diapause.

Finally we investigated the effects of diapause on the intestine. The diminished feeding and altered metabolism appear to affect the physiology and morphology of the midgut. Compared to one week old feeding control flies the midgut was significantly shorter during diapause and the length was restored after one week of recovery from 3 and 6 weeks of diapause. Our data are thus consistent with studies showing that growth of the gut is correlated with feeding level [48]. Using *NP1*-Gal4 driven GFP as a marker for gut epithelial cells and Hoechst staining for nuclei we showed here that the age-associated changes in the midgut are delayed by at least 3 weeks in diapausing flies. We note a slower deterioration of the epithelial cell layer and a delayed incidence of intestinal dysplasia throughout the lifespan of the diapausing flies compared to control flies. Intestinal dysplasia in aged flies may impact gut integrity and function [103], and eventually lead to chronic inflammation and death [104]. Slowed senescence of the intestine and prolonged lifespan during diapause could be connected to reduced feeding [105]. As was shown previously nutrient deprivation and reduced insulin-signaling in gut epithelial cells leads to diminished ISC proliferation [106], [107]. Reduced intestinal proliferation, low insulin-signaling and overexpression of stress protective genes regulated by FOXO are conditions known to improve proliferative homeostasis, delay degeneration and extend lifespan [108]. Low temperature conditions during diapause can play a role as well. In addition to a direct influence of temperature on the physiology of intestinal epithelial cells, low temperature can inhibit proliferation of gut microbiota, known to be deleterious in senescent flies [109]. For the future the intestine of diapausing flies would be an interesting system to investigate mechanisms of gut cell proliferation and aging.

Our study revealed that flies recover from extended diapause within a week of exposure to normal temperature and photoperiod. After one week of recovery from three weeks of diapause expression of all measured genes, except *Upd2*, returned to levels similar to those in one week old non-diapausing controls. In addition feeding was restored, ovaries developed, many of the metabolite levels returned to control levels, and the length of the intestine increased. Thus, with recovery during favorable conditions the flies are able to switch back to reproduction from the state of diapause-related somatic maintenance, in a display of adaptive regulatory plasticity (see [90]).

In summary, reproductive diapause in the adult fly involves dynamic alterations of multiple anabolic and catabolic pathways, peptide hormone levels, and innate immunity, and seems to have effects on several organs such as the endocrine system, ovaries, intestine and fat body. We also show that the flies recover from diapause within a week of return to normal temperature and day length, suggesting that the dormant stage can serve during periods of unfavorable conditions. The alterations in fuel metabolism and nutrient stores appear to shift dynamically from one state of equilibrium to another when entering diapause, and also during recovery. We propose that *D. melanogaster* with its short life cycle and genetic tractability, combined with its extensive use as a model in studies of metabolism and aging, is a favorable organism for further analysis of plasticity in adaptive life-history traits.

## Materials and Methods

### Fly husbandry and diapause induction

For the bulk of the experiments we used *Drosophila melanogaster* of the *Canton S* strain obtained from Bloomington Drosophila Stock Center (BDSC), Bloomington, IN). We also tested *D. melanogaster* with *dilp5*- or *dilp2-3* loss of function mutations (in *w^1118^* background) kindly provided by Sebastian Grönke (Cologne, Germany) and flies of the *w^1118^* strain (BDRC). For gut histology experiments the following lines were employed *yw;; UAS-mCD8-gfp* (III) from BDSC and w; *MyoIA-Gal4* (II) (*NP1-Gal4*, Jiang et al., 2009) was a kind gift from Ylva Engström (Stockholm University).

Parental flies were reared on BDSC food medium supplemented with 1.5 g/L nipagin (http://flystocks.bio.indiana.edu/Fly_Work/media-recipes/bloomfood.htm). For collection of eggs to be used for test flies 4–7-day old flies were transferred onto food medium containing 100 g/L sucrose, 50 g/L yeast, 12 g/L agar, 3 mL/L propionic acid and 3 g/L nipagin. Experimental flies were reared under uncrowded conditions at 25°C and normal photoperiod 12L:12D,. Newly eclosed 3–6–hour old virgin female flies were collected under mild CO_2_ anesthesia, put into 50 mL vials (10–15 flies per vial) containing 7 mL of the above mentioned medium and transferred to incubators with diapause conditions, 11°C and 10L:14D [13], [14]. Every three weeks during diapause treatment the food medium was changed. Diapausing flies were sampled every week during first three weeks and thereafter every three weeks until 12 weeks of diapause. Newly eclosed (3–6–hour old) virgin female flies were used as an initial control (Control 0 (C0)). Additionally, virgin flies, kept for 1 week at 25°C and normal photoperiod (12L:12D) were used as a control for non-diapausing flies [Control 1 (C1)]. To test the ability of flies to revert to normal metabolism and ovary development after diapause, flies were transferred to 25°C and 12L:12D after 3 and 6 weeks of diapause. These flies were kept for 1 week in normal conditions after 3-week diapause [Recovery 1′ (R1′)] and for 1 and 2 weeks after 6-week diapause [Recovery 1 (R1) and Recovery 2 (R2), respectively].

### Analysis of ovarian development

At each investigated time point of diapause (as well as in control and recovery groups) ovarian development was monitored microscopically and used as a main criterion for reproductive diapause as proposed by [13], [14], [41]. Ovaries were dissected in 0.01 M phosphate buffered saline (pH 7.4) buffer and their developmental stages assessed by using the combined criteria of King et al. [44], Saunders et al. [13], [41] and Shimada et al. [45]. Briefly, stages 1–7 of egg follicle development, covering previtellogenic stages, were divided into two subgroups with distinguishable oocytes (small previtellogenic ovaries, stage 2–5) and well recognizable separate oocytes), but without accumulated yolk (big previtellogenic ovaries, stage 6–7). Starting from small yolk deposits even in one single oocyte (stage 8) until one-third, one-half, and three quarters of the oocyte occupied by yolk (stages 9–11) ovaries were referred to as yolk accumulating group. The latter group together with ovaries of stages 12–14 with chorion formation and final maturity of the egg/eggs (several chorionated eggs) constitute vitellogenic ovaries. Results are expressed as percentage (%) of ovaries, in different development stages, or as incidence (%) of yolk accumulation in analyzed ovaries. Data are shown as means of 4 independent replicates with 8–12 flies in each replicate.

### Food intake assay

Food consumption was measured following the protocols of Skorupa et al. [47] and Wong el al. [46] with minor modifications. For analysis of food consumption 6–10 flies were allowed to feed for 6 h during daytime from the abovementioned sucrose-yeast medium (3 mL medium in 50 mL vial), supplemented with 0.5% FD & C Blue Dye no. 1 (Erioglaucine, Sigma-Aldrich). After feeding flies were frozen and stored at −80°C until further analysis. For each sample 6–10 flies were subjected to the same conditions in parallel, but without dye in the food (to control for any influence of eye pigment on the accuracy of the method) [46]. For analysis frozen flies were homogenized in centrifuge tubes in 50 mM phosphate buffer (pH 7.5) in the ratio 1∶20 (w/v) using a plastic pestle. Homogenates were centrifuged twice (16 000 g, 10 min, 25°C) and aliquots of final supernatants were used to measure absorbance at 629 nm [46] with a Genova spectrophotometer (Jenway, UK-PRC). The absorbance of samples from flies exposed to non-dyed food were used as baseline during spectrophotometry [46]. The amount food ingested was estimated from dye concentration by linear regression of data from a standard curve made with 0.2–4.0 µg erioglaucine. The amount of consumed food is expressed as nanograms of food ingested by 1 fly for 6 h (ng/fly/6 h).

#### Determination of carbohydrate concentrations in hemolymph and whole body

Virgin female flies of all investigated groups (control, diapause and recovery) and all four genotypes were used to measure concentrations of circulating (hemolymph) glucose and trehalose together with stored (whole body) glucose and trehalose as well as glycogen. For each sample 10–15 pre-weighed flies were decapitated and hemolymph was collected using a protocol modified after Broughton et al., and Demontis and Perrimon [110], [111], including centrifugation (3000 g, 4°C, for 6 min). Aliquots of hemolymph solution were kept for measurement of total protein concentration. The rest of hemolymph solutions were incubated for 5 min at 70°C to inactivate circulating enzymes [63], centrifuged (16 000 g, 4°C, 15 min) and deproteinized hemolymph supernatants were kept on ice until use. The pelleted fly bodies after hemolymph extraction were homogenized in PBS buffer (pH 7.4) in ratio 1∶10 (w/v) with subsequent centrifugation (16 000 g, 4°C, 15 min) and collection of supernatants. Aliquots of whole body supernatants were sampled for total protein measurement, while the remaining volumes were exposed to 70°C and centrifuged as above.

For cleavage of trehalose into glucose the aliquots of deproteinized hemolymph and whole body supernatants were incubated overnight at 37°C with 1.5 µl/mL porcine kidney trehalase (Sigma T8778). Glycogen from whole body supernatants was converted to glucose by overnight incubation at 37°C of supernatants with 0.5 mg/mL amyloglycosidase from *Aspergillus niger* (Sigma 10115).

All parameters were measured with a glucose assay kit with glucose oxidase and peroxidase (Liquick Cor-Glucose diagnostic kit, Cormay, Poland) following the manufacturer's guidelines. Absorbance of samples was measured at 500 nm with Genova (Jenway, UK-PRC) spectrophotometer. Glucose concentration was estimated with a linear regression coefficient from a standard curve made with 1.5–15 µg of glucose. Contents of trehalose and glycogen were calculated by subtracting free glucose concentration from the total glucose units measured. Concentrations of hemolymph glucose and trehalose are given in mM, whereas amount of body glucose and trehalose as well as glycogen are expressed as micrograms per milligram of wet mass of flies (µg/mgwm).

### Determination of total protein in hemolymph and body of flies

Total protein concentration in aliquots of non-deproteinized hemolymph and whole body supernatants, obtained as described above, was measured by the Coomassie blue method [112] using serum bovine albumin as a standard. Data are expressed either as micrograms of total protein per milliliter of hemolymph (µg/µL) or per gram wet mass of whole body (µg/gwm).

### Determination of content of triacylglycerides

For extraction of triacylglycerides (TAG) 10–15 pre-weighed flies per sample were homogenized in ratio 1∶10 (w/v) in PBS buffer (pH 7.4), supplemented with 0.05% Triton X. Homogenates were incubated for 15 min at 100°C, cooled on ice and centrifuged (16 000 g, 4°C, 10 min). Supernatants were collected and the amount of TAG was determined with a Liquick Cor-TG diagnostic kit (Cormay, Poland) using a linear regression coefficient from a standard curve made with 1.1–22 µg of TAG standard (Cormay, Poland). Absorbance of samples was measured at 550 nm with a spectrophotometer (Genova Jenway, UK-PRC). Data are expressed as micrograms of TAG per milligram of wet mass of flies (µg/mgwm)0.01 M.

### Morphometric analysis and imaging

10–15 virgin female flies from each of the investigated groups (control, diapause and recovery) and the four genotypes were weighted with a Mettler MT5 precision balance (Mettler Toledo AG, Switzerland) and the mean fly mass (mg) was calculated for each group. Data are presented from 8–10 independent replicates.

The abdomen size (mm^2^), midgut length and diameter (mm) and crop size (mm^2^) were analyzed in *Canton S* flies. Flies or dissected tissues were imaged with Leica Wild M32 microscope, connected with DF290 digital camera, and the parameters was analyzed using Leica Q Win and Image J software. Data shown represent mean values of 6–9 randomly selected flies for each replicate.

To visualize midgut epithelial cells we crossed *NP1-Gal4* flies [75] to *yw;; UAS-mCD8-gfp* (III) (BDSC). From these flies midguts (10 per replicate for each time point) were dissected in 0.01 M phosphate-buffered saline (PBS; pH 7.4), fixed in 4% paraformaldehyde in 0.1 M sodium phosphate buffer for 2 h and rinsed with 0.1% Triton-X in PBS (PBS-Tx) two times for 10 minutes. Nuclei/DNA were stained with Hoechst 33342 (1∶1000 dilution). These midguts were from w; *MyoIA-Gal4* (II) (*NP1-Gal4*, [75]) crossed to *yw;; UAS-mCD8-gfp* (III) from BDSC. Samples were washed with PBS-Tx three times and mounted in Fluoromount-G (SouthernBiotech) and analyzed on a Zeiss LSM 780 confocal microscope.

### Bacterial infection and antibiotics treatment of flies

Microbial infections of flies were performed with a mixture of over-night cultures of Gram-positive *Micrococcus luteus* and Gram-negative *Escherichia coli* which were washed once and suspended in 0.01 M PBS. Three weeks old flies previously kept either in diapause conditions or normal conditions were injected with <0.1 µL bacterial suspension per fly using a glass capillary connected to a micro injector (TriTech Research). After injection flies were transferred back to vials with sucrose-yeast medium and kept for an additional 3 hours before freezing at −80°C and RNA extraction. To exclude the possibility of some hidden infection or bacterial growth in medium we also kept part of the experimental flies on sucrose-yeast medium supplemented with antibiotics: 1 mg/ml carbancillin, 0.5 mg/ml vancomycin, 1 mg/ml neomycin and 0.168 mg/ml metronidazole [113].

### Quantitative real-time PCR (qPCR)

Total RNA was isolated from whole bodies of *Canton S* virgin female flies using Trizol-chloroform from four independent biological replicates with 10–15 flies in each replicate. cDNA was synthesized in triplicates, which were subsequently pooled together and diluted for qPCR. For cDNA synthesis reactions 2 µg of total RNA, 0.4 µL random hexamer primer (Thermo Scientific) and 2 µl of M-MuLV reversible transcriptase (Thermo Scientific) were used. The cDNA was then applied for quantitative real-time PCR (qPCR) using a Rotor Gene Q (Qiagen GmbH, Germany) instrument and SensiFAST SYBR Hi-ROX Kit (Bioline) as recommended by the manufacturer. For each sample triplicate reactions of the total volume of 20 µl were conducted with a primer concentration of 400 nM and 4 µl of diluted 1∶10 cDNA template. The mRNA levels were normalized to *rp49* levels in the same samples. Relative expression values were determined by the 2^−ΔΔCt^ method [114]. The sequences of the primers are shown in Table S1.

For transcription measurements of immune genes total RNA was extracted using Trizol reagent (Invitrogen) according to manufacturer's protocol and further purified by NucleoSpin RNA II kit (Macherey-Nagel) including an on-column digestion step with rDNase I. Quality and concentration of the RNA were determined with a NanoDrop 2000 spectrophotometer (Thermo Scientific). Total RNA (1000 ng) was applied for reverse transcription using the SuperScript III Reverse Transcriptase (Invitrogen) and oligo(dT)(20-mer). Quantitative PCR was performed comprising KAPA PROBE FAST Universal qPCR Master Mix (Kapa Biosystems) and the TaqMan Gene Expression Assays (Applied Biosystems) for the indicated genes, namely *PGRP-SB1* (Dm01805870_g1), or customized probes and the corresponding primers for *Drosomycin*, *Cecropin A1* and *Diptericin* [115]. The amplification was carried out in a Rotor-Gene Q (Qiagen). Each sample was analyzed in triplicate and normalized to the expression of *rp49* as an internal control.

### Data analysis

The experimental data are presented as means ± S.E.M. Statistical analysis was performed using R Statistical Software (Foundation for Statistical Computing, Vienna, Austria) version 3.0.3. Prior to statistical treatment all data were tested for homogeneity of variances using the Fligner-Killeen test and for normal distribution by the Shapiro-Wilk normality test. Unless otherwise stated statistical analysis was performed by one-way analysis of variance (ANOVA) followed by Tukey multiple comparisons test, when data had a normal distribution, and a non parametric Kruskal–Wallis test followed by pairwise comparisons using Wilcoxon rank sum test when data lacked normal distribution. The comparative analysis between fly strains under diapause and normal conditions and yolk accumulation incidence for all four strains were analyzed was made by unpaired Student t-test in the case of normally distributed data or Mann-Whitney-Wilcoxon rank sum test for data which did not display a normal distribution. A 95% confidence limit (P<0.05) was used throughout the study. Graphs were produced in OriginPro 7.5 software.

## Supporting Information

Table S1 **Primers used for quantitative PCR.**
(DOCX)

File S1 **Compressed Zip file of supporting figures.** **Fig. S1** Ovaries and intestinal structures are affected by diapause in *D. melanogaster* (*Canton S*). Dissected intestines with attached ovaries (asterisk) and crop (arrow) were imaged at the same magnification to reveal effects on diapause conditions. Typical images of five stages of flies are shown. **A** 3–6-h old control (C0). **B** One week old controls kept under normal conditions (C1). **C** Fly kept for 3 weeks of diapause (D3). **D** Fly after one week of recovery (R1′) after three weeks diapause. **E** A fly kept for 4 d at normal conditions, which represents a stage commonly used as a control. In **A1–D1** crops are shown at higher magnification. The crop is increased and translucent after three weeks of diapause (**C** and **C1**) and is opaque and smaller under non-diapausing conditions and recovery conditions (**B, B1** and **D, D1** and **E**). Ovaries are fully developed under normal conditions (**B**) and after recovery from diapause (**D**) but are previtellogenic in newly eclosed flies (**A**) and in flies in diapause (**E**). Note also that the midgut is opaque under normal and recovery conditions, whereas in newly eclosed flies and diapausing flies it is almost transparent. **Fig. S2** Transcript levels of immune genes in flies (*Canton S*) treated with antibiotics are similar to untreated flies. Analysis of (**A**) *Drosomycin*, (**B**) *Cecropin A1*, (**C**) *Peptidoglycan recognition proteins SB1* (*PGR-SB1*) and (**D**) *Diptericin* relative expression. Four of the fly groups shown in Fig. 7 are shown here with cross hatched bars representing flies fed a mixture of antibiotics (see results) and the others are untreated flies. The groups are 3–6 h old virgins (C0), controls kept one week in non-diapausing conditions (C1), flies kept for 3 weeks under non-diapausing (N3) and diapausing (D3) conditions. All these flies are non-infected. Data are presented as means ± S.E.M, *n* = 3–4 independent replicates with 10–15 flies in each. There are no significant differences between antibiotics-treated and non-treated flies (Student *t*-test). **Fig. S3** Ovarian development and yolk accumulation during diapause in *dilp5* and *dilp2-3* mutant flies. **A** and **B** Ovarian developmental stages (%) in virgin female *dilp5* (A) and *dilp2-3* (B) mutants, kept for 1–12 weeks at 11°C and short photoperiod 10L:14D, light/dark (diapause, D1–D12), or after recovery for 1 week after 3 weeks of diapause (R1′), 1 week (R1) or 2 weeks (R2) after 6 weeks of diapause. For comparison we used 3–6 h old virgin flies (C0), which have previtellogenic ovaries or virgin flies kept under normal conditions for 1 week (C1), which have fully developed ovaries. Criteria for ovarian developmental stages are described in *Materials and methods*. Data are presented as means ± S.E.M, *n* = 4 independent replicates with 8–12 flies in each replicate. See Fig. 1A and Fig. S7A in File S1 for ovarian development in *Canton S* and *w^1118^*, respectively. **C** and **D** Yolk accumulation incidence (%) in virgin *dilp5* (**C**) and *dilp2-3* (**D**) mutants kept under the same conditions as specified in Fig. S3A in File S1. Yolk accumulation incidence (%) was determined as availability of any yolk deposit even in a single oocyte (stage 8) up to formation of one/several chorionated eggs (stages 12–14) in mostly previtellogenic ovaries of diapausing flies. We indicate significance of differences to flies kept for one week at normal conditions (C1) that have 100% of yolk incidence, **p<0.01, ***p<0.001 (Student *t*-test). **E** Yolk accumulation incidence in all four fly lines tested (see Fig. 1B; Fig. S3C, D and Fig. S7B in File S1). These data demonstrate that the *dilp* mutants display by far the greatest reproductive diapause incidence, followed by *Canton S*, and with *w^1118^* as the least likely to diapause. **Fig. S4** Food intake and body mass are reduced during diapause in *dilp5* and *dilp2-3* mutant flies. **A** and **B** Food intake was measured in *dilp5* and *dilp2-3* mutant flies by feeding flies dyed food over 6 h at different time points during diapause (measured as µg/fly/6 h). Experimental conditions as in Fig. 2. We compared 3–6 h old flies (C0) flies kept for one week under non-diapause conditions (C1) to flies kept for 1–12 weeks under diapause conditions (D1–D12) and flies that recovered from diapause (R1′–R2). During diapause food intake decreases drastically compared to C1 flies. Food consumption is back to almost non-diapausing levels after recovery (R1′–2) from diapause. Data are presented as means ± S.E.M, *n* = 5–6 independent replicates with 6–10 flies in each replicate. We indicate significance values for experimental flies compared to the 1 week control (C1, grey bar) or as indicated by connectors. ***p<0.001 (ANOVA followed with Tukey test). **C** and **D** Body mass of whole flies was measured in flies corresponding to the sampling points in Fig. S4A and B in File S1. In mutants a significant decrease in body mass is seen already after one week of diapause (D1) and a gain is observed after recovery from 3 weeks of diapause (R1′). Data are presented as means ± S.E.M, *n* = 8–10 independent replicates with 10–15 flies in each replicate. We indicate significance values for experimental flies compared to C1 or as indicated by connectors, * *p*<0.05, ** *p*<0.01, ***p<0.001, N.S. not significantly different (ANOVA followed with Tukey test) or ^#^ *p*<0.05, ^##^ *p*<0.01 (Kruskal–Wallis test followed by pairwise comparisons using Wilcoxon rank sum test). **Fig. S5** Diapause conditions affect circulating and stored carbohydrates in *dilp5* and *dilp2-3* mutant flies. Here data for *dilp5* mutants are in the left column and *dilp2-3* in the right. Experimental conditions are as in Fig. S4 in File S1. Data significantly different from the flies kept for one week at normal conditions (C1) are indicated with * *p*<0.05, ** *p*<0.01, ***p<0.001 (ANOVA followed with Tukey test) or ^#^ *p*<0.05, ^##^ *p*<0.01, ^####^ *p*<0.01 (Kruskal–Wallis test followed by pairwise comparisons using Wilcoxon rank sum test), N.S. not significantly different. **A** and **B** Glucose concentration in hemolymph (mM) of female flies kept under control or diapausing conditions, as well as flies recovered from diapause, as specified in Fig. 2and Fig. S4 in File S1. Compared to one week old controls (C1) the glucose levels at 3–12 weeks of diapause (D3–D12) are higher. After recovery from 6 weeks of diapause (R1) glucose levels increase further in *dilp5* mutants, but not for *dilp2-3* mutants. **C** and **D** Trehalose levels in the hemolymph increase significantly during diapause (D1–D12) in *dilp2-3* mutants, but not in *dilp5* mutants. **E** and **F** Whole body glucose increases significantly during diapause in both mutants, and remains high after recovery, compared with flies, kept under normal conditions (C1). Newly eclosed flies (C0) display significantly higher body glucose than C1 flies only in the *dilp5* line. **G** and **H** Whole body trehalose is not affected in *dilp5* mutants, but increases significantly to a peak at three weeks diapause (D3) and return to the control level (C1) during recovery. **Fig. S6** Diapause conditions affect levels of glycogen, triacylglycerides and protein in *dilp5* and *dilp2-3* mutant flies. Experimental conditions are as in Fig. S4 in File S1. Data are presented as means ± S.E.M, *n* = 5–6 independent replicates with 10–15 flies in each replicate and statistics as in Fig. S5 in File S1. **A** and **B** In both mutants flies kept for one week under normal conditions (C1) store glycogen than newly eclosed flies (C0), or 1-week old flies, kept under diapause conditions (D1). In *dilp5*-deficient flies glycogen stores increase during diapause and peak at 2–3 weeks of diapause (D2–D3) the decrease during extended diapause (D9–D12) compared to non-diapausing control flies (C1), whereas in the double mutant glycogen stores at D2 and D3 do not exceed that at C1. **C** and **D** Triacylglyceride (TAG) contents also increase during diapause in both mutants compared to non-diapausing controls (C1). The *dilp5*-deficient flies, that recovered from diapause, still maintain higher TAG contents than C1, but this is not observed for *dilp2-3*-defficient flies. **E** and **F** In both mutants the total protein in the hemolymph drops significantly in the 1-week old control flies, (C1) compared to newly eclosed controls (C0), but increases in flies kept for one week under diapausing conditions (D1). The *dilp2-3* mutant shows enhanced levels of circulating protein throughout diapause and recovery, whereas in the *dilp5* mutant changes are smaller. **G** and **H** The total body protein is slightly decreased throughout diapause and recovery in *dilp2-3* mutants in compared to non-diapausing flies (C1), but it is more variable in *dilp5* mutants. Furthermore, the 3–4-h old flies (C0) display higher body protein in *dilp5* flies compared to C1 flies, whereas in *dilp2-3* mutants there is no difference between C0 and C1. **Fig. S7** Diapause phenotypes observed in *w^1118^* strain of *D. melanogaster*. Virgin female *w^1118^* flies were kept under the same conditions as described in Fig. S4 in File S1. Data are presented as means ± S.E.M, *n* = 5–6 independent replicates with 10–15 flies in each replicate. Statistics as specified in Fig. S3 (for yolk accumulation incidence data) and Fig. S5 (for metabolite levels) in File S1. **A** Ovarian developmental stages (%) in *w^1118^* flies. **B** Incidence of yolk accumulation (%) in virgin female *w^1118^* flies. *w^1118^* flies seem to be less prone to reproductive diapause, since yolk accumulation in ovaries increases throughout diapause without a clear stage of fully arrested vitellogenesis. **C** Food intake in flies diapausing for one week (D1) is drastically decreased compared to one week controls (C1) and corresponds to that in newly eclosed flies (C0). The food consumption recovered to normal levels after diapause (R1′, R1–R2). **D** The fly mass decreases significantly in flies under diapause conditions (D1–D12) and it does not recover after diapause (R1′, R1–R2) compared to non-diapausing flies (C1). **E** Hemolymph glucose increases during diapause and remains enhanced after recovery compared to the control flies (C1). **F** Trehalose in the hemolymph increases drastically during the first two weeks of diapause (D1–D2) compared to non-diapausing flies (C1), but diapause (D3–D12) and recovery (R1′, R1–R2) diminish circulating trehalose to the level of controls (C1). **G** The whole body glucose concentrations are significantly elevated over 2–12 weeks of diapause (D2–D12) compared to C1 controls, and after recovery (R1′, R1–R2) they remain elevated. **H** The whole body trehalose levels are significantly elevated over 12 weeks of diapause compared to non-diapausing controls (C1) and diminish after recovery (R1′, R1–R2). **I** Glycogen stores are variable during diapause with peaks at 2–3 weeks (D2–D3) and then diminish below control (C1) levels. **J** TAG content is elevated throughout diapause and does not decrease during recovery compared to the C1 control. **K** Total protein in the hemolymph is also variable, but mostly elevated during diapause and recovery. **L** Total protein in the body is diminished throughout diapause and recovery. In the above experiments the newly eclosed flies (C0) differ from the 1-week-old control flies, kept at non-diapausing conditions (C1) in the following way: the C0 flies display higher level of circulating protein (K), but not glucose (E) and trehalose (F); in contrast stored glucose (G) and glycogen (I), as well as body protein (L) are more accumulated in C1 flies than in C0, while stored trehalose (H) and TAG content (J) is higher in newly eclosed flies (C0). **Fig. S8** The mortality is negligible during diapause in all *D. melanogaster* strains tested. We calculated mortality in the four *D. melanogaster* lines and found that over 12 weeks of diapause they all followed a similar profile with a mortality below 20% until 9 weeks diapause and then around 30% at 12 weeks. Flies kept under normal non-diapausing conditions do not survive until 12 weeks, and after 9 weeks less than 20% of the flies survive [21].
(ZIP)
