# Supplementary material for: The Sleeping Beauty: How Reproductive Diapause Affects Hormone Signaling, Metabolism, Immune Response and Somatic Maintenance in Drosophila melanogaster
Source: PLoS One. 2014 Nov 13;9(11):e113051. doi: 10.1371/journal.pone.0113051 (PMC4231144; doi:10.1371/journal.pone.0113051)
Supplement: Table S1 — Primers used for quantitative PCR. (DOCX) [file pone.0113051.s001.docx]

Table S1

***Primers for qPCR (all displayed 5’–3’)***

*Akh* F: AGACCTCCAACGAAATGCTG

*Akh* R: GTGCTTGCAGTCCAGAAAGAG

*dilp2* F: AGCAAGCCTTTGTCCTTCATCTC

*dilp2* R: ACACCATACTCAGCACCTCGTTG;

*dilp3* F: TGTGTGTATGGCTTCAACGCAATG

*dilp3* R: CACTCAACAGTCTTTCCAGCAGGG;

*dilp5* F: GAGGCACCTTGGGCCTATTC

*dilp5* R: CATGTGGTGAGATTCGG

*dilp6* F: CCCTTGGCGATGTATTTCCCAACA

*dilp6* R: CCGACTTGCAGCACAAATCGGTTA

*InR F*: ACTGAACCTCTCGTCAAGGC

*InR R*: GAACCCTCCACGCACTTACA

*tobi* F: CCACCAAGCGAGACATTTACC

*tobi* R: GAGCGGCGTAGTCCATCAC

*Upd2* F: CGGAACATCACGATGAGCGAAT

*Upd2* R: TCGGCAGGAACTTGTACTCG

*4ebp* F: CCAGGAAGGTTGTCATCTCG

*4ebp* R: CCAGGAGTGGTGGAGTAGAGG

*PEPCK* F: TCAATGGCGAATCCTGCTAC

*PEPCK* R: TCCTTCACGTCCACCTTATCC’

*rp49* F: ATCGGTTACGGATCGAACAA

*rp49* R: GACAATCTCCTTGCGCTTCT
