# Supplementary figures and images for: The Sleeping Beauty: How Reproductive Diapause Affects Hormone Signaling, Metabolism, Immune Response and Somatic Maintenance in Drosophila melanogaster
Source: PLoS One. 2014 Nov 13;9(11):e113051. doi: 10.1371/journal.pone.0113051 (PMC4231144; doi:10.1371/journal.pone.0113051)

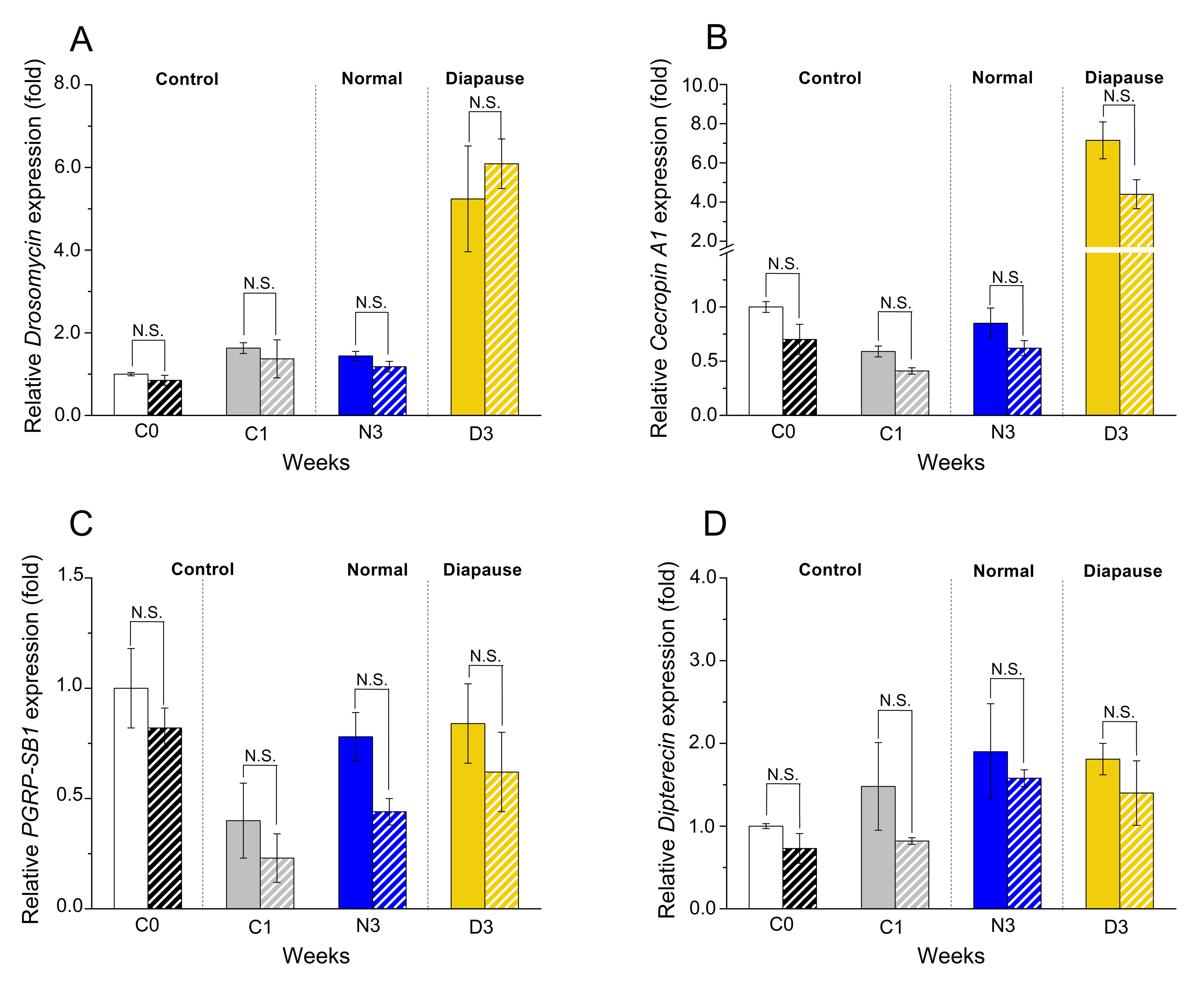

Supplement: File S1 — Compressed Zip file of supporting figures. Fig. S1 Ovaries and intestinal structures are affected by diapause in D. melanogaster (Canton S). Dissected intestines with attached ovaries (asterisk) and crop (arrow) were imaged at the same magnification to reveal effects on diapause conditions. Typical images of five stages of flies are shown. A 3–6-h old control (C0). B One week old controls kept under normal conditions (C1). C Fly kept for 3 weeks of diapause (D3). D Fly after one week of recovery (R1′) after three weeks diapause. E A fly kept for 4 d at normal conditions, which represents a stage commonly used as a control. In A1–D1 crops are shown at higher magnification. The crop is increased and translucent after three weeks of diapause (C and C1) and is opaque and smaller under non-diapausing conditions and recovery conditions (B, B1 and D, D1 and E). Ovaries are fully developed under normal conditions (B) and after recovery from diapause (D) but are previtellogenic in newly eclosed flies (A) and in flies in diapause (E). Note also that the midgut is opaque under normal and recovery conditions, whereas in newly eclosed flies and diapausing flies it is almost transparent. Fig. S2 Transcript levels of immune genes in flies (Canton S) treated with antibiotics are similar to untreated flies. Analysis of (A) Drosomycin, (B) Cecropin A1, (C) Peptidoglycan recognition proteins SB1 (PGR-SB1) and (D) Diptericin relative expression. Four of the fly groups shown in Fig. 7 are shown here with cross hatched bars representing flies fed a mixture of antibiotics (see results) and the others are untreated flies. The groups are 3–6 h old virgins (C0), controls kept one week in non-diapausing conditions (C1), flies kept for 3 weeks under non-diapausing (N3) and diapausing (D3) conditions. All these flies are non-infected. Data are presented as means ± S.E.M, n = 3–4 independent replicates with 10–15 flies in each. There are no significant differences between antibiotics-treated and [file pone.0113051.s002.zip › Fig. S1-8/Fig. S2.tif]

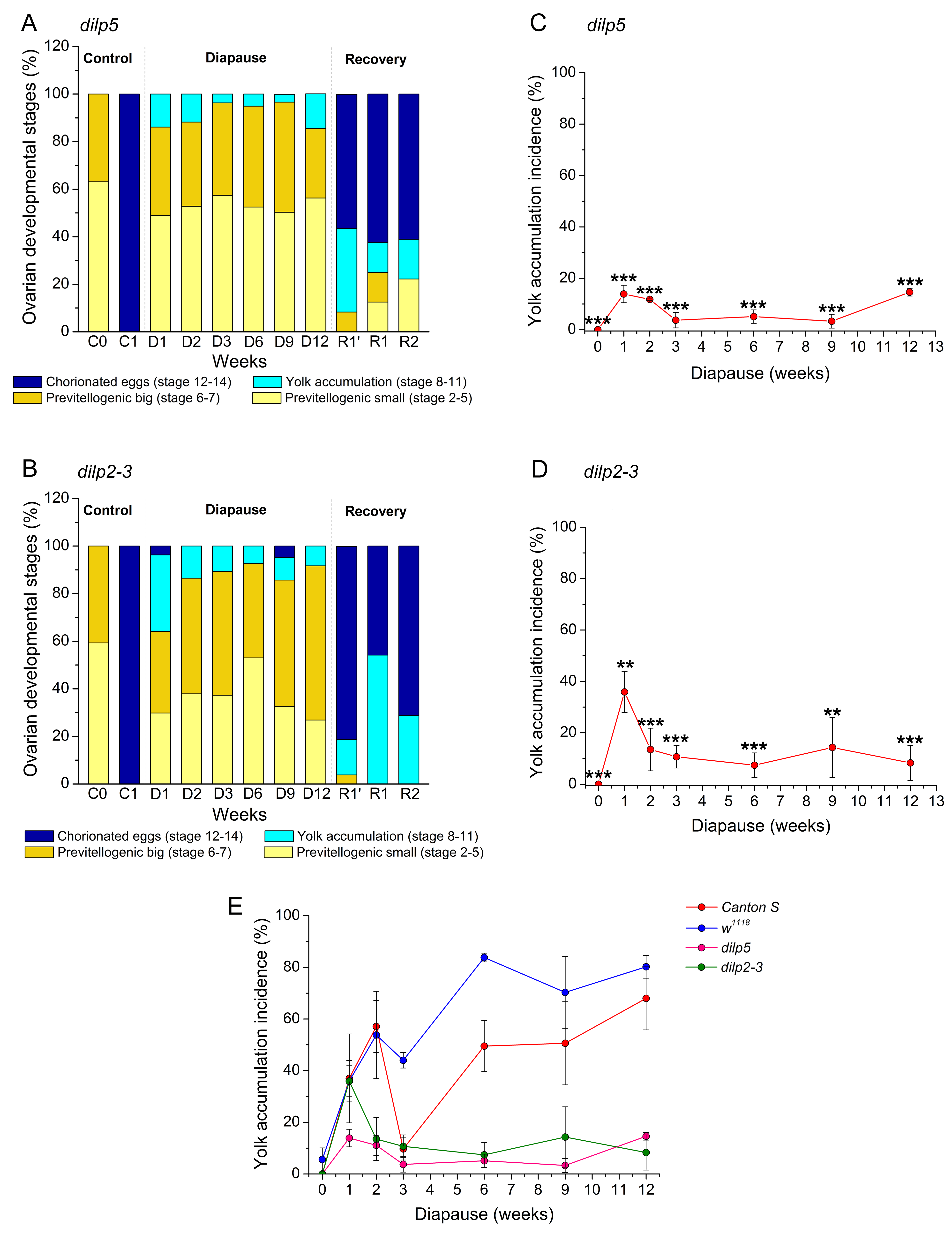

Supplement: File S1 — Compressed Zip file of supporting figures. Fig. S1 Ovaries and intestinal structures are affected by diapause in D. melanogaster (Canton S). Dissected intestines with attached ovaries (asterisk) and crop (arrow) were imaged at the same magnification to reveal effects on diapause conditions. Typical images of five stages of flies are shown. A 3–6-h old control (C0). B One week old controls kept under normal conditions (C1). C Fly kept for 3 weeks of diapause (D3). D Fly after one week of recovery (R1′) after three weeks diapause. E A fly kept for 4 d at normal conditions, which represents a stage commonly used as a control. In A1–D1 crops are shown at higher magnification. The crop is increased and translucent after three weeks of diapause (C and C1) and is opaque and smaller under non-diapausing conditions and recovery conditions (B, B1 and D, D1 and E). Ovaries are fully developed under normal conditions (B) and after recovery from diapause (D) but are previtellogenic in newly eclosed flies (A) and in flies in diapause (E). Note also that the midgut is opaque under normal and recovery conditions, whereas in newly eclosed flies and diapausing flies it is almost transparent. Fig. S2 Transcript levels of immune genes in flies (Canton S) treated with antibiotics are similar to untreated flies. Analysis of (A) Drosomycin, (B) Cecropin A1, (C) Peptidoglycan recognition proteins SB1 (PGR-SB1) and (D) Diptericin relative expression. Four of the fly groups shown in Fig. 7 are shown here with cross hatched bars representing flies fed a mixture of antibiotics (see results) and the others are untreated flies. The groups are 3–6 h old virgins (C0), controls kept one week in non-diapausing conditions (C1), flies kept for 3 weeks under non-diapausing (N3) and diapausing (D3) conditions. All these flies are non-infected. Data are presented as means ± S.E.M, n = 3–4 independent replicates with 10–15 flies in each. There are no significant differences between antibiotics-treated and [file pone.0113051.s002.zip › Fig. S1-8/Fig. S3.tif]

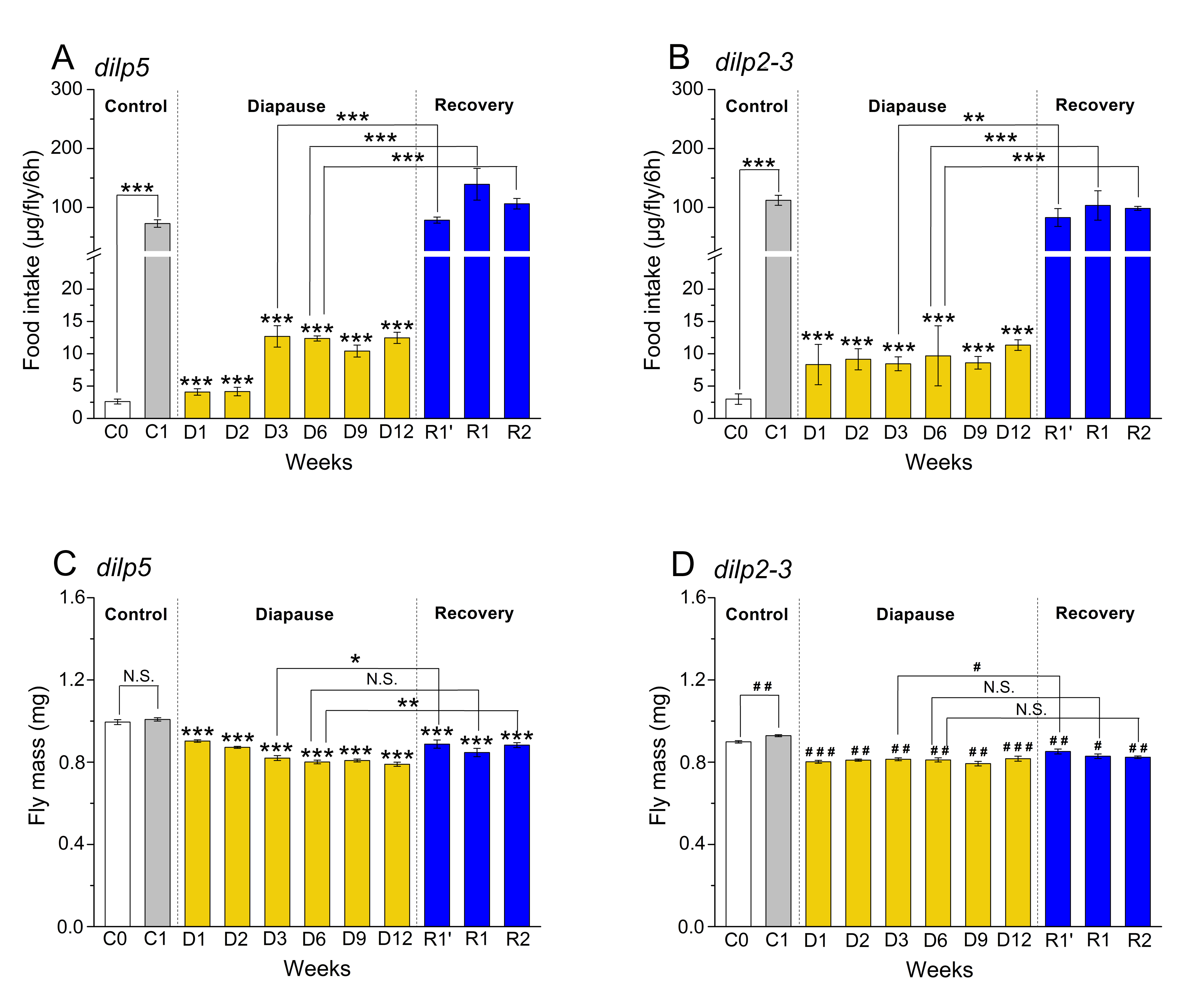

Supplement: File S1 — Compressed Zip file of supporting figures. Fig. S1 Ovaries and intestinal structures are affected by diapause in D. melanogaster (Canton S). Dissected intestines with attached ovaries (asterisk) and crop (arrow) were imaged at the same magnification to reveal effects on diapause conditions. Typical images of five stages of flies are shown. A 3–6-h old control (C0). B One week old controls kept under normal conditions (C1). C Fly kept for 3 weeks of diapause (D3). D Fly after one week of recovery (R1′) after three weeks diapause. E A fly kept for 4 d at normal conditions, which represents a stage commonly used as a control. In A1–D1 crops are shown at higher magnification. The crop is increased and translucent after three weeks of diapause (C and C1) and is opaque and smaller under non-diapausing conditions and recovery conditions (B, B1 and D, D1 and E). Ovaries are fully developed under normal conditions (B) and after recovery from diapause (D) but are previtellogenic in newly eclosed flies (A) and in flies in diapause (E). Note also that the midgut is opaque under normal and recovery conditions, whereas in newly eclosed flies and diapausing flies it is almost transparent. Fig. S2 Transcript levels of immune genes in flies (Canton S) treated with antibiotics are similar to untreated flies. Analysis of (A) Drosomycin, (B) Cecropin A1, (C) Peptidoglycan recognition proteins SB1 (PGR-SB1) and (D) Diptericin relative expression. Four of the fly groups shown in Fig. 7 are shown here with cross hatched bars representing flies fed a mixture of antibiotics (see results) and the others are untreated flies. The groups are 3–6 h old virgins (C0), controls kept one week in non-diapausing conditions (C1), flies kept for 3 weeks under non-diapausing (N3) and diapausing (D3) conditions. All these flies are non-infected. Data are presented as means ± S.E.M, n = 3–4 independent replicates with 10–15 flies in each. There are no significant differences between antibiotics-treated and [file pone.0113051.s002.zip › Fig. S1-8/Fig. S4.tif]

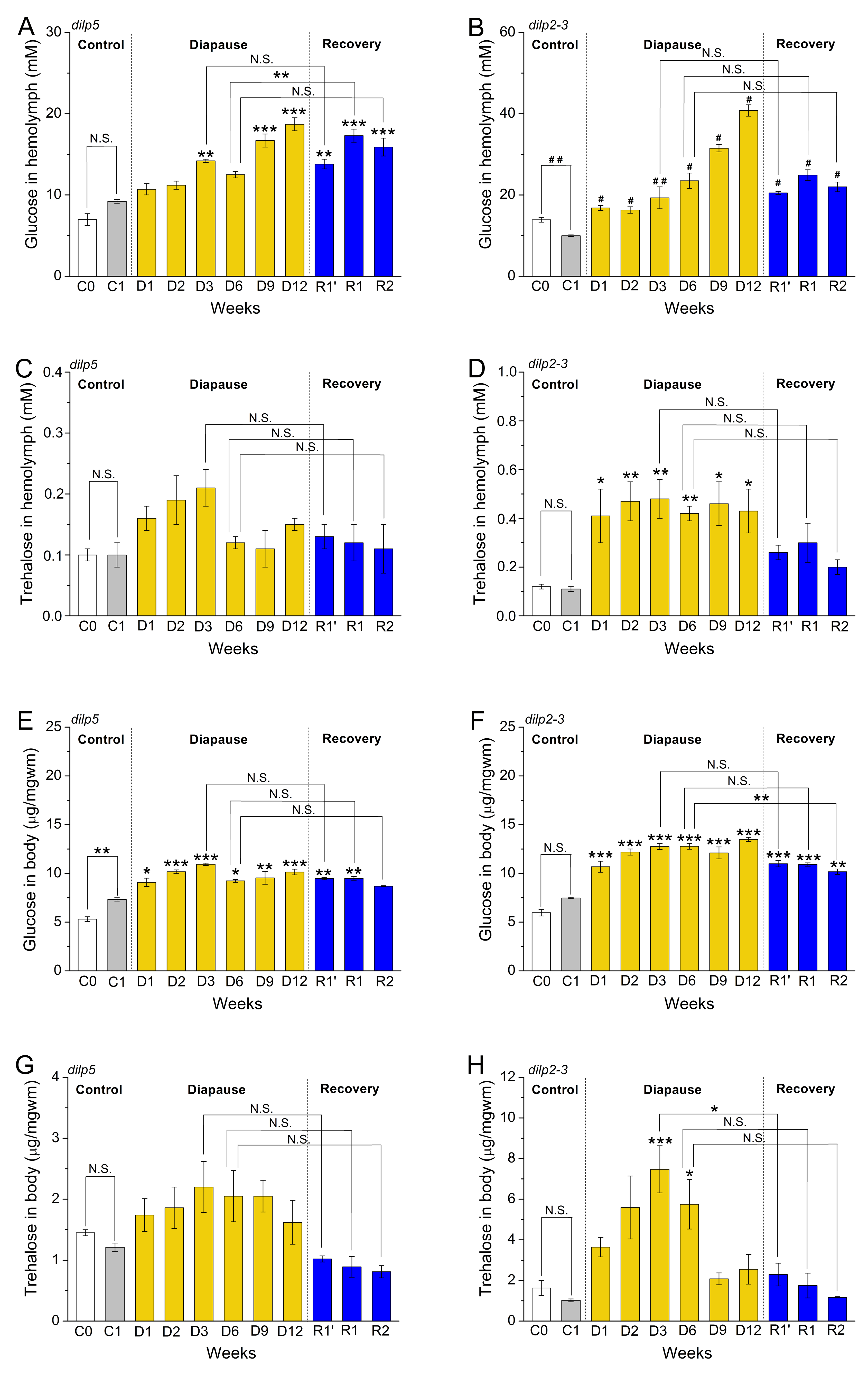

Supplement: File S1 — Compressed Zip file of supporting figures. Fig. S1 Ovaries and intestinal structures are affected by diapause in D. melanogaster (Canton S). Dissected intestines with attached ovaries (asterisk) and crop (arrow) were imaged at the same magnification to reveal effects on diapause conditions. Typical images of five stages of flies are shown. A 3–6-h old control (C0). B One week old controls kept under normal conditions (C1). C Fly kept for 3 weeks of diapause (D3). D Fly after one week of recovery (R1′) after three weeks diapause. E A fly kept for 4 d at normal conditions, which represents a stage commonly used as a control. In A1–D1 crops are shown at higher magnification. The crop is increased and translucent after three weeks of diapause (C and C1) and is opaque and smaller under non-diapausing conditions and recovery conditions (B, B1 and D, D1 and E). Ovaries are fully developed under normal conditions (B) and after recovery from diapause (D) but are previtellogenic in newly eclosed flies (A) and in flies in diapause (E). Note also that the midgut is opaque under normal and recovery conditions, whereas in newly eclosed flies and diapausing flies it is almost transparent. Fig. S2 Transcript levels of immune genes in flies (Canton S) treated with antibiotics are similar to untreated flies. Analysis of (A) Drosomycin, (B) Cecropin A1, (C) Peptidoglycan recognition proteins SB1 (PGR-SB1) and (D) Diptericin relative expression. Four of the fly groups shown in Fig. 7 are shown here with cross hatched bars representing flies fed a mixture of antibiotics (see results) and the others are untreated flies. The groups are 3–6 h old virgins (C0), controls kept one week in non-diapausing conditions (C1), flies kept for 3 weeks under non-diapausing (N3) and diapausing (D3) conditions. All these flies are non-infected. Data are presented as means ± S.E.M, n = 3–4 independent replicates with 10–15 flies in each. There are no significant differences between antibiotics-treated and [file pone.0113051.s002.zip › Fig. S1-8/Fig. S5A-H.tif]

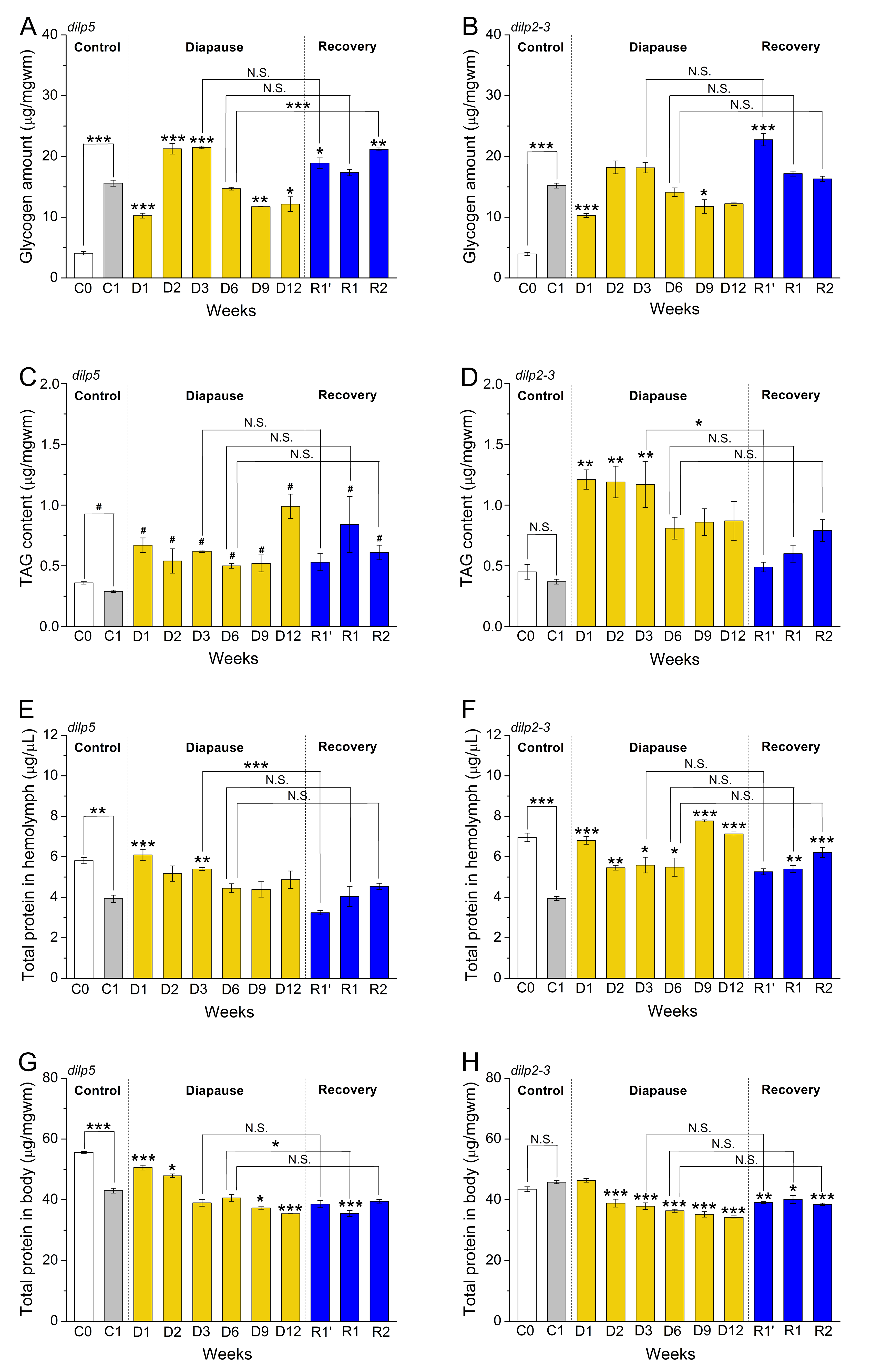

Supplement: File S1 — Compressed Zip file of supporting figures. Fig. S1 Ovaries and intestinal structures are affected by diapause in D. melanogaster (Canton S). Dissected intestines with attached ovaries (asterisk) and crop (arrow) were imaged at the same magnification to reveal effects on diapause conditions. Typical images of five stages of flies are shown. A 3–6-h old control (C0). B One week old controls kept under normal conditions (C1). C Fly kept for 3 weeks of diapause (D3). D Fly after one week of recovery (R1′) after three weeks diapause. E A fly kept for 4 d at normal conditions, which represents a stage commonly used as a control. In A1–D1 crops are shown at higher magnification. The crop is increased and translucent after three weeks of diapause (C and C1) and is opaque and smaller under non-diapausing conditions and recovery conditions (B, B1 and D, D1 and E). Ovaries are fully developed under normal conditions (B) and after recovery from diapause (D) but are previtellogenic in newly eclosed flies (A) and in flies in diapause (E). Note also that the midgut is opaque under normal and recovery conditions, whereas in newly eclosed flies and diapausing flies it is almost transparent. Fig. S2 Transcript levels of immune genes in flies (Canton S) treated with antibiotics are similar to untreated flies. Analysis of (A) Drosomycin, (B) Cecropin A1, (C) Peptidoglycan recognition proteins SB1 (PGR-SB1) and (D) Diptericin relative expression. Four of the fly groups shown in Fig. 7 are shown here with cross hatched bars representing flies fed a mixture of antibiotics (see results) and the others are untreated flies. The groups are 3–6 h old virgins (C0), controls kept one week in non-diapausing conditions (C1), flies kept for 3 weeks under non-diapausing (N3) and diapausing (D3) conditions. All these flies are non-infected. Data are presented as means ± S.E.M, n = 3–4 independent replicates with 10–15 flies in each. There are no significant differences between antibiotics-treated and [file pone.0113051.s002.zip › Fig. S1-8/Fig. S6A-H.tif]

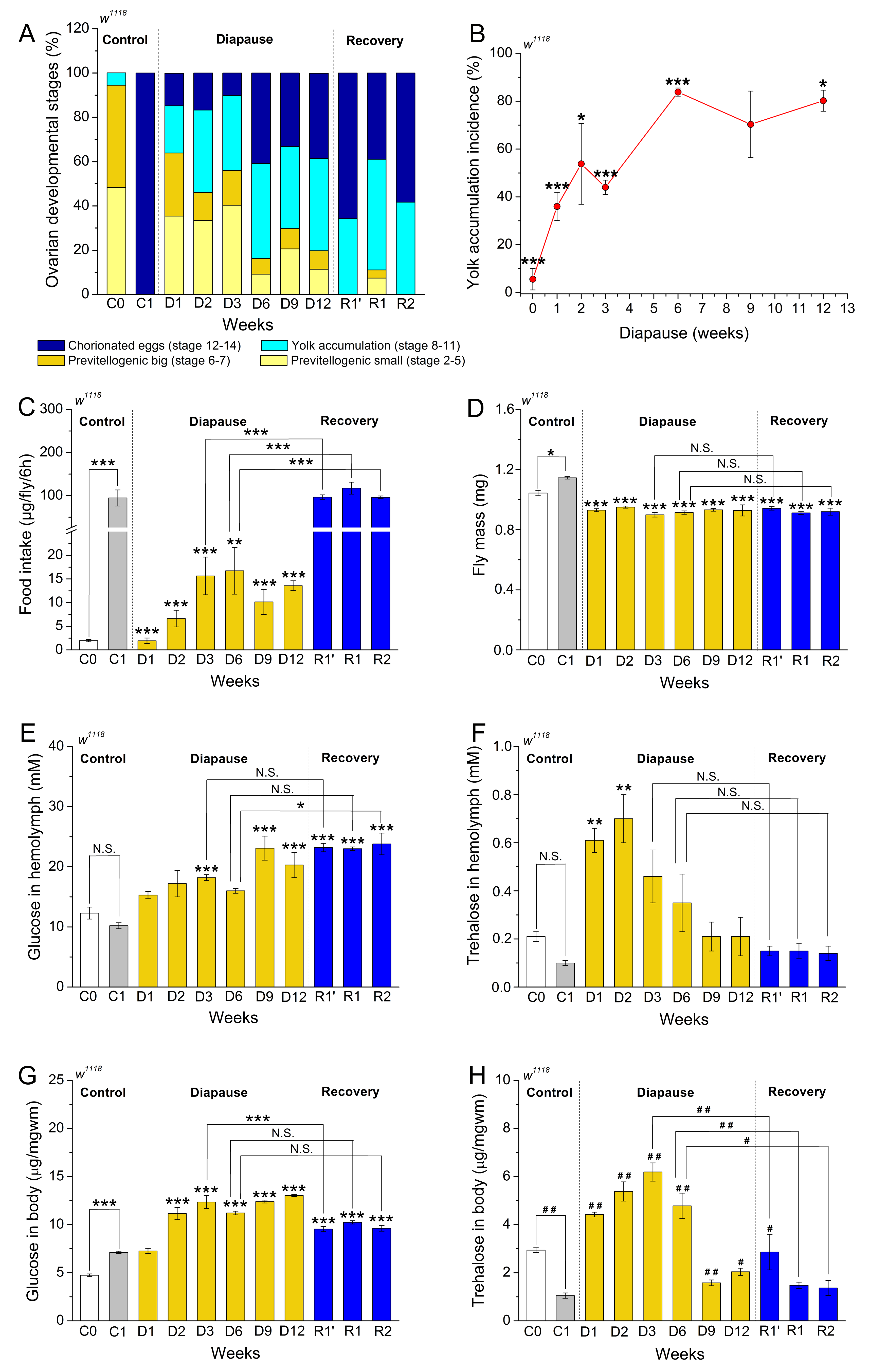

Supplement: File S1 — Compressed Zip file of supporting figures. Fig. S1 Ovaries and intestinal structures are affected by diapause in D. melanogaster (Canton S). Dissected intestines with attached ovaries (asterisk) and crop (arrow) were imaged at the same magnification to reveal effects on diapause conditions. Typical images of five stages of flies are shown. A 3–6-h old control (C0). B One week old controls kept under normal conditions (C1). C Fly kept for 3 weeks of diapause (D3). D Fly after one week of recovery (R1′) after three weeks diapause. E A fly kept for 4 d at normal conditions, which represents a stage commonly used as a control. In A1–D1 crops are shown at higher magnification. The crop is increased and translucent after three weeks of diapause (C and C1) and is opaque and smaller under non-diapausing conditions and recovery conditions (B, B1 and D, D1 and E). Ovaries are fully developed under normal conditions (B) and after recovery from diapause (D) but are previtellogenic in newly eclosed flies (A) and in flies in diapause (E). Note also that the midgut is opaque under normal and recovery conditions, whereas in newly eclosed flies and diapausing flies it is almost transparent. Fig. S2 Transcript levels of immune genes in flies (Canton S) treated with antibiotics are similar to untreated flies. Analysis of (A) Drosomycin, (B) Cecropin A1, (C) Peptidoglycan recognition proteins SB1 (PGR-SB1) and (D) Diptericin relative expression. Four of the fly groups shown in Fig. 7 are shown here with cross hatched bars representing flies fed a mixture of antibiotics (see results) and the others are untreated flies. The groups are 3–6 h old virgins (C0), controls kept one week in non-diapausing conditions (C1), flies kept for 3 weeks under non-diapausing (N3) and diapausing (D3) conditions. All these flies are non-infected. Data are presented as means ± S.E.M, n = 3–4 independent replicates with 10–15 flies in each. There are no significant differences between antibiotics-treated and [file pone.0113051.s002.zip › Fig. S1-8/Fig. S7A-H.tif]

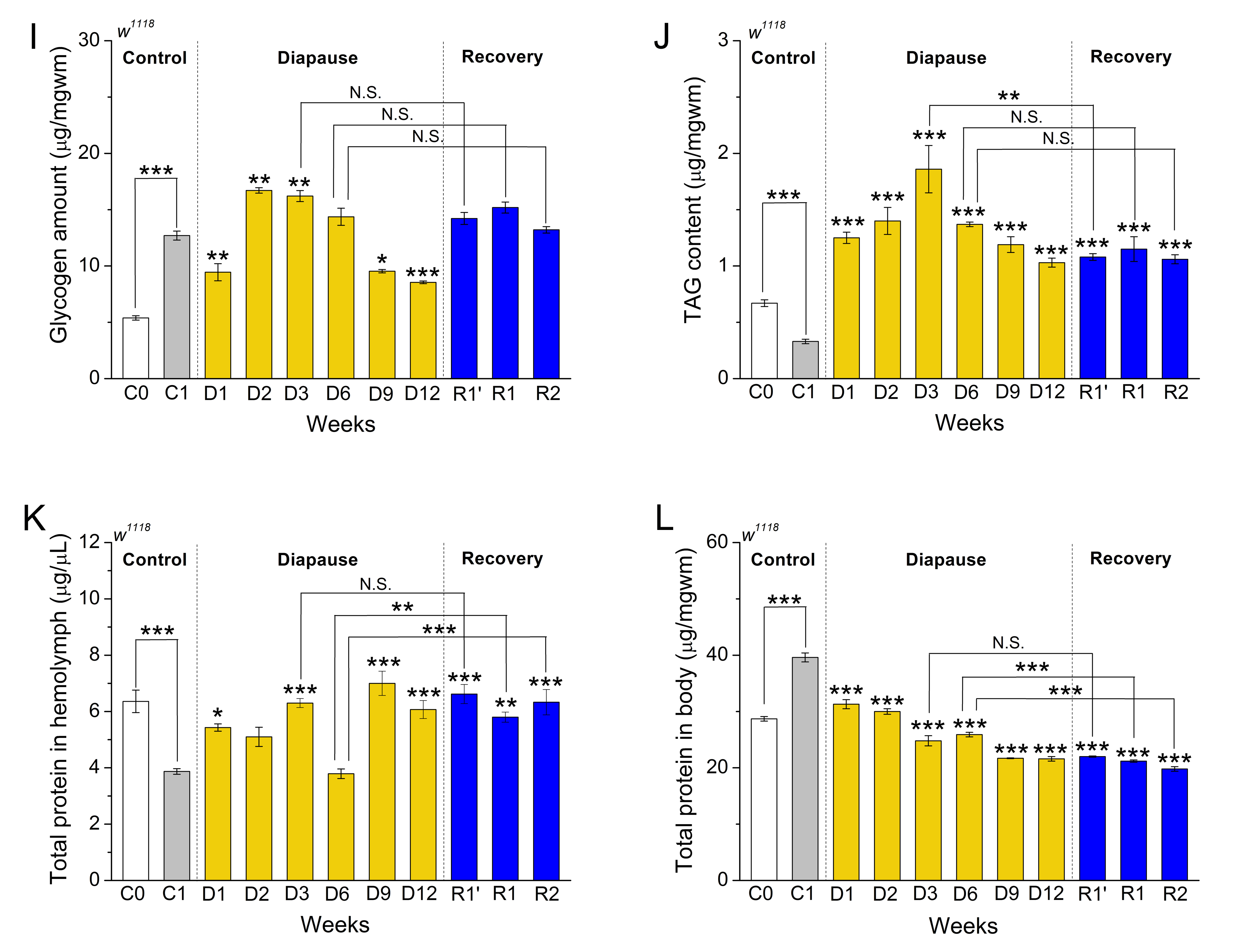

Supplement: File S1 — Compressed Zip file of supporting figures. Fig. S1 Ovaries and intestinal structures are affected by diapause in D. melanogaster (Canton S). Dissected intestines with attached ovaries (asterisk) and crop (arrow) were imaged at the same magnification to reveal effects on diapause conditions. Typical images of five stages of flies are shown. A 3–6-h old control (C0). B One week old controls kept under normal conditions (C1). C Fly kept for 3 weeks of diapause (D3). D Fly after one week of recovery (R1′) after three weeks diapause. E A fly kept for 4 d at normal conditions, which represents a stage commonly used as a control. In A1–D1 crops are shown at higher magnification. The crop is increased and translucent after three weeks of diapause (C and C1) and is opaque and smaller under non-diapausing conditions and recovery conditions (B, B1 and D, D1 and E). Ovaries are fully developed under normal conditions (B) and after recovery from diapause (D) but are previtellogenic in newly eclosed flies (A) and in flies in diapause (E). Note also that the midgut is opaque under normal and recovery conditions, whereas in newly eclosed flies and diapausing flies it is almost transparent. Fig. S2 Transcript levels of immune genes in flies (Canton S) treated with antibiotics are similar to untreated flies. Analysis of (A) Drosomycin, (B) Cecropin A1, (C) Peptidoglycan recognition proteins SB1 (PGR-SB1) and (D) Diptericin relative expression. Four of the fly groups shown in Fig. 7 are shown here with cross hatched bars representing flies fed a mixture of antibiotics (see results) and the others are untreated flies. The groups are 3–6 h old virgins (C0), controls kept one week in non-diapausing conditions (C1), flies kept for 3 weeks under non-diapausing (N3) and diapausing (D3) conditions. All these flies are non-infected. Data are presented as means ± S.E.M, n = 3–4 independent replicates with 10–15 flies in each. There are no significant differences between antibiotics-treated and [file pone.0113051.s002.zip › Fig. S1-8/Fig. S7I-L.tif]

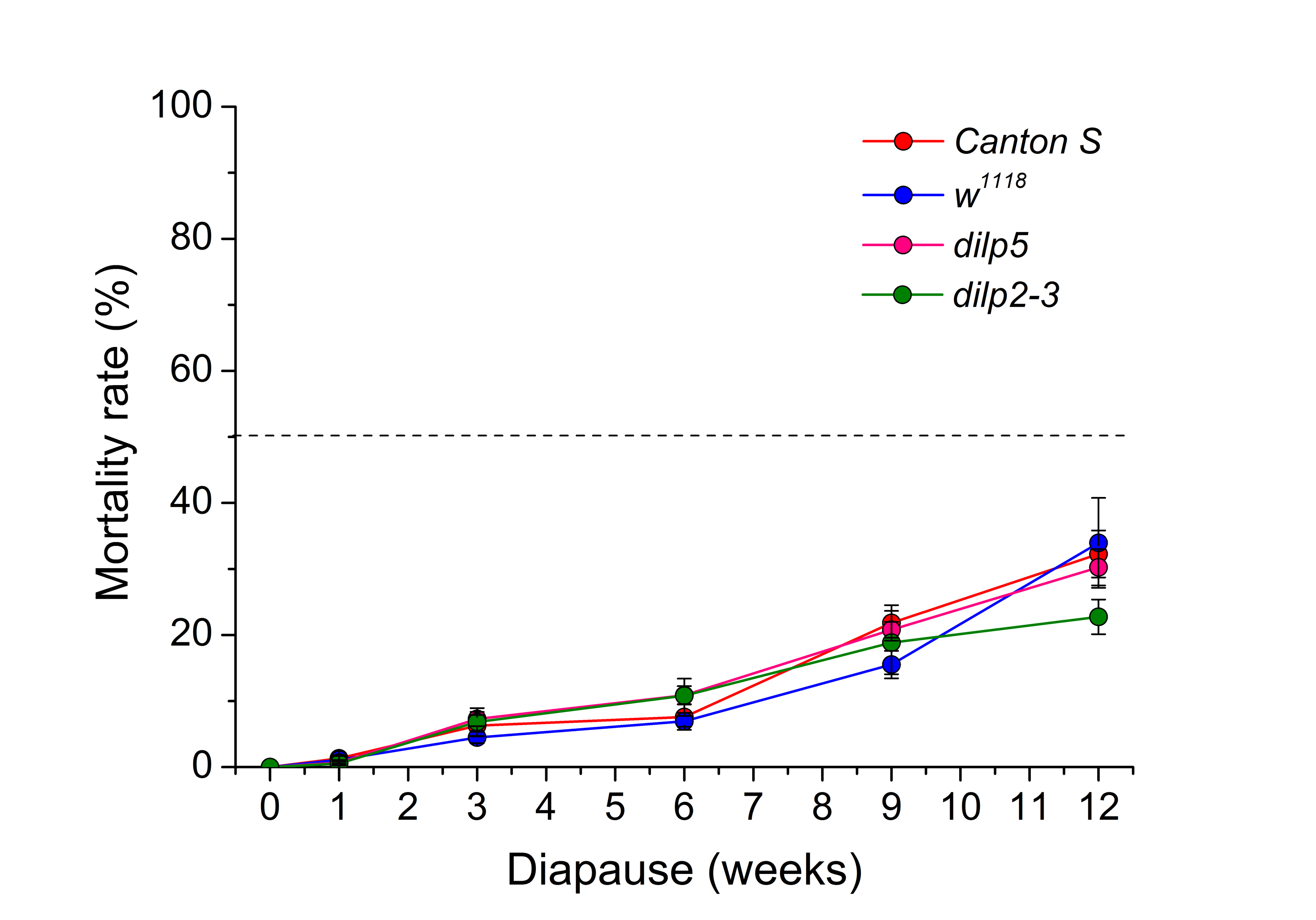

Supplement: File S1 — Compressed Zip file of supporting figures. Fig. S1 Ovaries and intestinal structures are affected by diapause in D. melanogaster (Canton S). Dissected intestines with attached ovaries (asterisk) and crop (arrow) were imaged at the same magnification to reveal effects on diapause conditions. Typical images of five stages of flies are shown. A 3–6-h old control (C0). B One week old controls kept under normal conditions (C1). C Fly kept for 3 weeks of diapause (D3). D Fly after one week of recovery (R1′) after three weeks diapause. E A fly kept for 4 d at normal conditions, which represents a stage commonly used as a control. In A1–D1 crops are shown at higher magnification. The crop is increased and translucent after three weeks of diapause (C and C1) and is opaque and smaller under non-diapausing conditions and recovery conditions (B, B1 and D, D1 and E). Ovaries are fully developed under normal conditions (B) and after recovery from diapause (D) but are previtellogenic in newly eclosed flies (A) and in flies in diapause (E). Note also that the midgut is opaque under normal and recovery conditions, whereas in newly eclosed flies and diapausing flies it is almost transparent. Fig. S2 Transcript levels of immune genes in flies (Canton S) treated with antibiotics are similar to untreated flies. Analysis of (A) Drosomycin, (B) Cecropin A1, (C) Peptidoglycan recognition proteins SB1 (PGR-SB1) and (D) Diptericin relative expression. Four of the fly groups shown in Fig. 7 are shown here with cross hatched bars representing flies fed a mixture of antibiotics (see results) and the others are untreated flies. The groups are 3–6 h old virgins (C0), controls kept one week in non-diapausing conditions (C1), flies kept for 3 weeks under non-diapausing (N3) and diapausing (D3) conditions. All these flies are non-infected. Data are presented as means ± S.E.M, n = 3–4 independent replicates with 10–15 flies in each. There are no significant differences between antibiotics-treated and [file pone.0113051.s002.zip › Fig. S1-8/Fig. S8.tif]

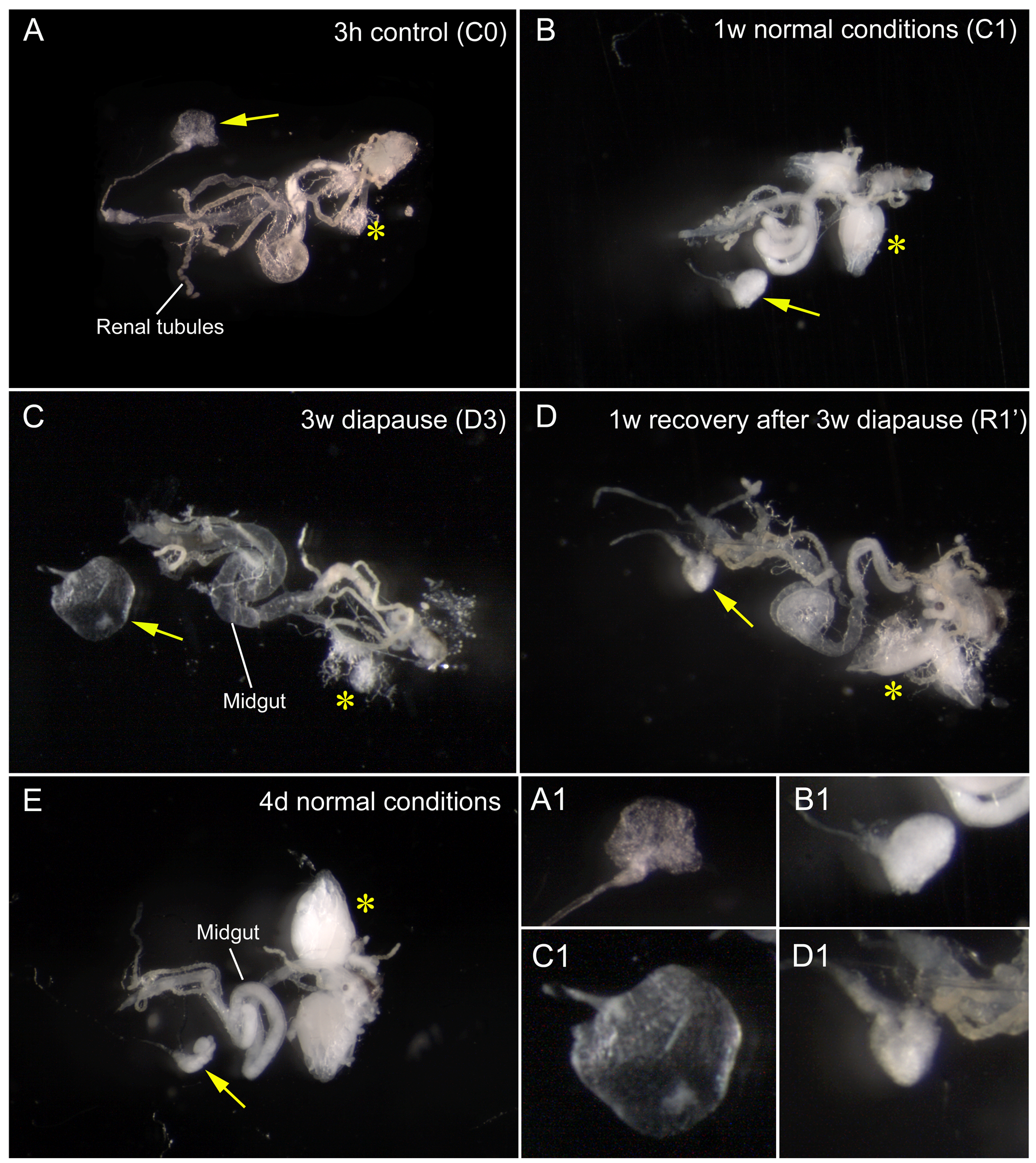

Supplement: File S1 — Compressed Zip file of supporting figures. Fig. S1 Ovaries and intestinal structures are affected by diapause in D. melanogaster (Canton S). Dissected intestines with attached ovaries (asterisk) and crop (arrow) were imaged at the same magnification to reveal effects on diapause conditions. Typical images of five stages of flies are shown. A 3–6-h old control (C0). B One week old controls kept under normal conditions (C1). C Fly kept for 3 weeks of diapause (D3). D Fly after one week of recovery (R1′) after three weeks diapause. E A fly kept for 4 d at normal conditions, which represents a stage commonly used as a control. In A1–D1 crops are shown at higher magnification. The crop is increased and translucent after three weeks of diapause (C and C1) and is opaque and smaller under non-diapausing conditions and recovery conditions (B, B1 and D, D1 and E). Ovaries are fully developed under normal conditions (B) and after recovery from diapause (D) but are previtellogenic in newly eclosed flies (A) and in flies in diapause (E). Note also that the midgut is opaque under normal and recovery conditions, whereas in newly eclosed flies and diapausing flies it is almost transparent. Fig. S2 Transcript levels of immune genes in flies (Canton S) treated with antibiotics are similar to untreated flies. Analysis of (A) Drosomycin, (B) Cecropin A1, (C) Peptidoglycan recognition proteins SB1 (PGR-SB1) and (D) Diptericin relative expression. Four of the fly groups shown in Fig. 7 are shown here with cross hatched bars representing flies fed a mixture of antibiotics (see results) and the others are untreated flies. The groups are 3–6 h old virgins (C0), controls kept one week in non-diapausing conditions (C1), flies kept for 3 weeks under non-diapausing (N3) and diapausing (D3) conditions. All these flies are non-infected. Data are presented as means ± S.E.M, n = 3–4 independent replicates with 10–15 flies in each. There are no significant differences between antibiotics-treated and [file pone.0113051.s002.zip › Fig. S1-8/Fig. S1.tif]
